# Cleavable Cationic Carbosilane Dendrimers with pH-Tunable Charge as siRNA Carriers

**DOI:** 10.1021/acs.biomac.5c00344

**Published:** 2025-07-18

**Authors:** Judith Recio-Ruiz, Paulina Rycharska, Małgorzata Grygiel, Sylwia Michlewska, Maria Bryszewska, Maksim Ionov, Francisco Javier de la Mata, Sandra García-Gallego

**Affiliations:** † 16720 University of Alcala, Department of Organic and Inorganic Chemistry and Research Institute in Chemistry “Andrés M. Del Río” (IQAR), Madrid 28805, Spain; ‡ 49602 University of Lodz, Faculty of Biology and Environmental Protection, Department of General Biophysics, Pomorska 141/143, Lodz 90-236, Poland; § BioMedChem Doctoral School of the University of Lodz and Lodz Institutes of the Polish Academy of Sciences. Matejki 21/23, Lodz 90-237, Poland; ∥ 49602 University of Lodz, Faculty of Biology and Environmental Protection, Laboratory of Microscopic Imaging and Specialized Biological Techniques, Banacha 12/16, Lodz 90-237, Poland; ⊥ Faculty of Medicine, Collegium Medicum, Mazovian Academy in Plock, Pl. Dabrowskiego 2, Plock 09-402, Poland; # Networking Research Center on Bioengineering, Biomaterials and Nanomedicine (CIBER-BBN), Madrid 28029, Spain; ∇ Institute Ramón y Cajal for Health Research (IRYCIS), Madrid 28034, Spain

## Abstract

Gene therapy is a cutting-edge technique for the prevention or treatment of diseases, which demands the development of biocompatible and efficient vectors. Additionally, for cancer therapy, a precise control on the vectors’ response at different pH values is required due to the heterogeneity of the tumors’ pH. Many nonviral vectors are based on quaternary ammonium compounds, thus exhibiting potential toxicity and persistence. In this work, we designed a new family of cationic carbosilane dendrimers, which exhibits two differential features: a pH-tunable charge, which can modulate the interaction between the nanocarrier and the siRNA, and a cleavable core, which can further enhance the release of the cargo. We thoroughly explored the structural and biophysical characteristics of the new dendrimers at different pH values: 4.5, 5.5, and 7.4. Additionally, biocompatibility in tumoral and nontumoral cells was evaluated along with the hemolytic effect. Finally, we provided a proof-of-concept for their use as pH-responsive nanocarriers of siRNA.

## Graphical abstract

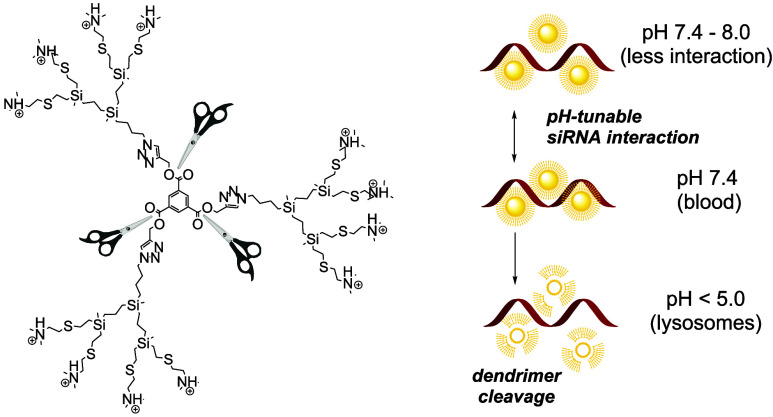

## 1 Introduction

Gene therapy is a cutting-edge technique, which employs genetic material such as DNA, mRNA, or siRNA for the prevention or treatment of both inherited and acquired diseases.^1^ For the genetic material to be preserved until reaching the target place due to the extreme instability and low cellular uptake level, nanocarriers are generally required for effective gene therapy. Nonviral vectors present several advantages over viral counterparts, such as low immunogenicity and the possibility of a large-scale production, but typically they exhibit lower efficacy.^2^

Nanotechnology offers innovative tools to improve the therapeutic efficacy of nonviral vectors.^3^ In particular, dendrimers and monodisperse and hyperbranched polymeric scaffolds have been broadly studied as gene carriers.^4^ Dendrimers can be surface-engineered on demand, thus overcoming extracellular and intracellular barriers and improving gene delivery.^5,6^ A myriad of functional groups have been used for dendrimer modification, including lipids, amino acids, saccharides, peptides, or, very frequently, cationic groups like oligo amine, tertiary amine, quaternary ammonium, imidazolium, guanidium, and phosphonium.^5^ In particular, quaternary ammonium compounds (QACs) exhibited promising features as gene carriers^7^ and have been used in many consumer applications including antimicrobials or surfactants. Their permanently charged structure allows interactions with negatively charged bacterial cell membranes, leading to efficient membrane disruption and bacterial death. Nevertheless, it has been reported that high QAC exposure can lead to toxicity and significant health risks.^8^

For gene therapy applications, the vector’s biocompatibility is crucial. In general, dendrimer cytotoxicity depends on the generation, the number of surface groups, and the nature of terminal moieties (anionic, neutral, or cationic). Higher cytotoxicity is observed for higher-generation and positively charged dendrimers.^9^ Accordingly, additional groups like polyethylene glycol (PEG) chains, acetyl groups, carbohydrates, and other moieties have been included in the multivalent scaffold to decrease the cytotoxicity.^5^ However, an optimal balance between interactions with nucleic acids and cell toxicity must be reached in order to obtain an optimal vector performance.

Carbosilane dendrimers have been broadly studied as gene carriers.^10^ These highly stable, lipophilic dendritic scaffolds composed of carbon–carbon and carbon–silicon bonds are functionalized with cationic groups, mainly quaternary ammonium (NMe_3_^+^), in order to increase the solubility in water and facilitate electrostatic interactions with negatively charged nucleic acids. Numerous studies with quaternized carbosilane dendrimers confirmed the successful delivery of siRNA and ODN to different cells in HIV^11,12^ and cancer^13,14^ models. Importantly, cationic carbosilane dendrimers can transfect both adherent and suspension cell lines, with the latter considered as resistant to common synthetic vectors.^15^ They promote the cellular uptake by interactions with hydrophobic and hydrophilic regions of the cell membrane.^16^

Nevertheless, the strong electrostatic interaction between quaternary ammonium groups and the anionic nucleic acid sometimes hinders the release of the cargo, leading to a low therapeutic efficiency.^14^ To solve this problem, different strategies have been proposed such as PEGylation,^17^ the buried-charge approach,^18^ or the use of nonpermanent cationic charge. In the latter, a second-generation carbosilane dendrimer with a hydroquinone core was functionalized with 8 units of either −NMe_2_HCl or −NMe_3_I to explore potential differences between a pH-dependent or a permanent cationic charge. The authors confirmed that despite both dendrimers transported siRNA into the cell, the dendrimer bearing −NMe_2_HCl was more promising as an siRNA carrier in MCF-7 cells cultured as 3D spheroids. The siRNA complex with the −NMe_3_I dendrimer aggregated into larger particles, resulting in a lower penetration in the spheroid and a lower cytotoxicity.^19^ This exemplifies the potential of dendrimers with a pH-tunable charge.

Furthermore, in cancer treatment, the pH is a relevant parameter to consider. It has been well described that the tumors’ microenvironment exhibits acidic properties due to the high metabolic activity and insufficient perfusion, showing around 0.3–0.7 pH units lower than the average extracellular pH of normal tissues.^20^ For example, extracellular pH switched from 7.4 (skin) to 7.0 (melanoma); 8.0 (vulvar) to 7.3 (vulvar tumor); or 7.3 (lung) to 6.4 (lung tumor).^21^ These differences could enable a selective therapy toward tumor tissues. Surprisingly, it has also been reported that the average intracellular pH of tumor cells is neutral or slightly alkaline, thus establishing relevant differences between intra- and extracellular pH, for example, from 7.2 to 6.4 for lung tumor. In conclusion, tumor pH is spatially and temporally heterogeneous and demands a precise control of the response of potential vectors at different pH values, mainly in the range of 6.0–8.0.

Herein, we designed a new family of cationic carbosilane dendrimers with pH-tunable charge and an additional feature: cleavability. At physiological pH, the dendrimers are fully protonated, exhibiting strong interactions with nucleic acids. A subtle increase in pH decreases the electrostatic interaction, which improves the cargo release. Furthermore, at a pH below 5.0, cleavage of the dendrimer core can further enhance the release of nucleic acids. We present the structural and biophysical characterization of the new dendrimers and a proof-of-concept for their use as pH-responsive nanocarriers of siRNA.

## 2 Experimental Section

Comprehensive details of the materials and methods used in this work are described in the Supporting Information (ESI). Synthetic protocols toward dendrimers **1**–**3** are described below. The structure and purity of **1**–**3** were confirmed via ^1^H, ^13^C, and 2D-NMR (using a Bruker Neo400 spectrometer), elemental analysis, MALDI-TOF, DLS, and zeta potential measurements.

### 2.1 Synthesis and Characterization of Cleavable Carbosilane Dendrimers

#### 2.1.1 General Procedure to Synthesize ArGn­(NMe_2_HCl)_m_ Dendrimers

Precursor dendrimers **ArGnV**_**m**_ (**I**–**III)** were synthesized as previously reported.^22^ The dendrimer **ArGnV**_**m**_ and 2-dimethylaminoethanethiol hydrochloride (1.1 equiv/alkene) were dissolved in THF/MeOH. The photoinitiator DMPA (5% mol/alkene) was added. The reaction mixture was stirred gently until complete dissolution of the reagents and then exposed to UV light (365 nm, 30 W) for 4 h. After completion of the reaction, it was dialyzed in methanol until the complete removal of byproducts.

#### 2.1.2 ArG1­(NMe_2_HCl)_6_ (**1**)

Dendrimer **1** was synthesized through the general procedure using the following reagents: ArG1V_6_ (394.7 mg, 0.433 mmol), 2-dimethylaminoethanethiol hydrochloride (368.3 mg, 2.60 mmol), and DMPA (33.3 mg, 0.130 mmol).

^1^H NMR (400 MHz, MeOD): δ 8.80 (s, 3H, Ar–H^Ph^), 8.19 (s, 3H, Ar–H^triazole^), 5.51 (s, 6H, −COOCH_2_−), 4.46 (m, 6H, N^triazole^CH_2_−), 3.33 (t, 12H, −CH_2_NMe_2_·HCl), 3.33 (t, 6H, −CH_2_NMe_2_·HCl), 2.90 (t, 12H, −SCH_2_CH_2_N−), 2.90 (36H, −NCH_3_), 2.65 (t, 12H, −SiCH_2_CH_2_S−), 1.95 (m, 6H, N^triazole^CH_2_CH_2_−), 1.32 (m, 6H, N^triazole^CH_2_CH_2_CH_2_−), 0.91 (t, 12H, −SiCH_2_CH_2_S−), 0.68 (m, 6H, N^triazole^(CH_2_)_3_CH_2_−), 0.12 (s, 9H, −SiCH_3_). ^13^C NMR (400 MHz, MeOD): δ 164.8 (COO), 142.3 (C^triazole^), 135.2 (Ar–CH^Ph^), 131.1 (Ar–C^Ph^), 124.1 (CH^triazole^), 59.4 (−COOCH_2_−), 57.9 (NCH_2_CH_2_S), 53.1 (NCH_2_), 50.8 (NMe_2_·HCl), 43.7 (NCH_3_), 34.3 (NCH_2_CH_2_), 26.9 (NCH_2_CH_2_S), 21.4 (−CH_2_CH_2_Si−), 15.2 (−CH_2_CH_2_Si−), 13.6 (−CH_2_Si−), −5.33 (−SiCH_3_). C_69_H_135_Cl_6_N_15_O_6_S_6_Si_3_ (1760.25 g/mol). Calcd %C 47.08; %H 7.73; %N 11.94. Exp. %C 47.00; %H 8.01; %N 11.54. *m*/*z* calcd 1547.6, exp. 1540.6 Da.

#### 2.1.3 ArG2­(NMe_2_HCl)_12_ (**2**)

Dendrimer **2** was synthesized through the general procedure using the following reagents: ArG2V_12_ (131.1 mg, 0.083 mmol), 2-dimethylaminoethanethiol hydrochloride (141.1 mg, 0.996 mmol), and DMPA (12.8 mg, 0.050 mmol).

^1^H NMR (400 MHz, MeOD): δ 8.71 (s, 3H, Ar–H^Ph^), 8.16 (s, 3H, Ar–H^triazole^), 5.48 (s, 6H, −COOCH_2_−), 4.43 (m, 6H, NCH_2_−), 3.33 (t, 24H, −CH_2_NMe_2_·HCl), 3.33 (t, 12H, −CH_2_NMe_2_·HCl), 2.90 (t, 24H, −SCH_2_CH_2_−), 2.90 (72H, NCH_3_), 2.65 (t, 24H, −SCH_2_CH_2_−), 1.32 (m, 6H, N^triazole^CH_2_CH_2_−), 0.85 (m, 18H, −SCH_2_CH_2_Si−), 0.60–0.50 (m, 54H, −CH_2_Si−), 0.10 (s, 18H, −SiCH_3_), −0.12 (s, 9H, −SiCH_3_). ^13^C NMR (400 MHz, MeOD): δ 164.8 (COO), 138.8 (C^triazole^), 135.2 (Ar–CH^Ph^), 131.1 (Ar–C^Ph^), 124.1 (CH^triazole^), 59.4 (−COOCH_2_−), 57.9 (NCH_2_CH_2_S), 53.1 (NCH_2_), 50.8 (NMe_2_·HCl), 43.7 (NCH_3_), 34.3 (NCH_2_CH_2_), 26.9 (NCH_2_CH_2_S), 21.4 (−CH_2_CH_2_Si−), 15.2 (−CH_2_CH_2_Si−), 13.6 (−CH_2_Si−), −5.33 (−SiCH_3_). C_129_H_279_Cl_12_N_21_O_6_S_12_Si_9_ (3283.68 g/mol). Calcd %C 47.19; %H 8.56; %N 8.96. Exp. %C 47.12; %H 8.55; %N 8.72.

#### 2.1.4 ArG3­(NMe_2_HCl)_24_ (**3**)

Dendrimer **3** was synthesized through the general procedure using the following reagents: ArG3V_24_ (81.5 mg, 0.028 mmol), 2-dimethylaminoethanethiol hydrochloride (95.2 mg, 0.672 mmol), and DMPA (8.6 mg, 0.034 mmol).

^1^H NMR (400 MHz, MeOD): 8.81 (s, 3H, Ar–H^Ph^), 8.25 (s, 3H, Ar–H^triazole^), 5.51 (s, 6H, −COOCH_2_−), 4.50 (t, 6H, NCH_2_−), 3.33 (t, 48H, −CH_2_NMe_2_·HCl), 3.33 (t, 24H, −CH_2_NMe_2_·HCl), 2.90 (s, 48H, −SCH_2_CH_2_−), 2.90 (s, 144H, NCH_3_), 2.70 (t, 48H, −SCH_2_CH_2_−), 1.92 (m, 6H, N^triazole^CH_2_CH_2_−), 1.40 (m, 42H, N^triazole^CH_2_CH_2_CH_2_−), 0.85 (t, 48H, −SCH_2_CH_2_Si−), 0.70–0.50 (m, 78H, −CH_2_Si−), 0.15 (s, 36H, −SiCH_3_). ^13^C NMR (400 MHz, MeOD): δ 164.8 (COO), 142.3 (C^triazole^), 135.2 (Ar–CH^Ph^), 131.1 (Ar–C^Ph^), 124.1 (CH^triazole^), 59.4 (−COOCH_2_−), 57.9 (NCH_2_CH_2_S), 53.1 (NCH_2_), 50.8 (NMe_2_·HCl), 43.7 (NCH_3_), 34.3 (NCH_2_CH_2_), 26.9 (NCH_2_CH_2_S), 21.4 (−CH_2_CH_2_Si−), 15.2 (−CH_2_CH_2_Si−), 13.6 (−CH_2_Si−), −5.33 (−SiCH_3_). C_249_H_567_Cl_24_N_33_O_6_S_24_Si_21_ (6330.52 g/mol). Calcd %C 47.24; %H 9.03; %N 7.30. Exp. %C 47.20; %H 8.92; %N 7.24.

### 2.2 Potentiometric Study

Dendrimers were dissolved in distilled water (0.5 mg/mL) and brought to pH 12 using a 0.1 M NaOH solution. Subsequently, aliquots of the 0.1 M HCl solution were gradually added, and the pH of the solution was recorded after each addition. p*K*_a_ values were calculated through the second derivative method and compared to predicted values obtained through MarvinSketch 22.7.

### 2.3 Degradation Kinetics Study

Dendrimer degradation was monitored through ^1^H NMR and DOSY assays in a deuterated buffered medium, adjusted to pH 7.4, 5.5, or 4.5, and maintained at a temperature of either 25 or 37 °C. Evidence of compound degradation was detected by signal broadening and the appearance of new peaks in the spectra.

### 2.4 Hydrodynamic Diameter and Zeta Potential Measurements

The particle size and distribution were evaluated using a Zetasizer Nano ZS spectrometer (Malvern Instruments Ltd., UK). Measurements were performed using three buffer solutions at pH 4.5, 5.5, and 7.4 and different time points (0, 1.5, 4, and 24 h). Dendrimers were dissolved in the corresponding buffer solution at a concentration of 5 mM. For experiments with siRNA, the siLuc3 siRNA was used in a 0.3 μM concentration. Prior to measurements, siRNA was incubated with dendrimers for 15 min at 24 °C and pH 7.4.

### 2.5 PBMC Isolation and Cell Cultures

PBMCs were isolated from buffy coats obtained from the Central Blood Bank, Lodz, Poland. Blood was diluted 1:1 with PBS, placed on a Histopaque gradient, and centrifuged. Cells were washed three times with PBS and then erythrocyte lysing buffer was added. Prewarmed RPMI 1640 supplemented with 10% FBS and 1% antibiotics was added, and cells were counted and seeded onto a 96-well plate at a density of 10.000/well. Cells were kept in a humid atmosphere (37 °C, 5% CO_2_).

The THP-1 cell line (human leukemia monocytic cell line) was cultured in RPMI 1640 supplemented with 10% FBS and 1% penicillin/streptomycin in 37 °C and 5% CO_2_. To assess cytotoxicity, cells were seeded onto 96-well black plates at a density of 10.000 cells per well. Cytotoxicity assay was performed after 24 h of incubation.

The HEK-293 cell line (human embryonic kidney cells) was cultured in a DMEM medium supplemented with 10% FBS and 1% penicillin/streptomycin. Cells were seeded onto a 96-well plate at a density of 10.000 cells per well, incubated for 24 h, and then treated with the compounds.

### 2.6 Cytotoxicity Assays

Dendrimer solutions were prepared in 5 mM phosphate buffer at increasing concentrations in the range of 0.1–10 μM, using buffers at different pH values (4.5, 5.5, 7.4) and incubated for 3 h at r.t. before cell treatment. Cells were incubated with dendrimers for 24 h.

#### 2.6.1 MTT Assay

HEK 293T cell cytotoxicity was evaluated using the MTT assay. MTT solution was added to the final concentration of 0.5 mg/mL. After 2 h incubation at 37 °C and 5% CO_2_, MTT solution was discarded and 100 μL of DMSO was added to dissolve formazan crystals. The absorbance was measured at λ_ex_ = 570 nm and λ_em_ = 720 nm using a Synergy HTX plate reader.

#### 2.6.2 Alamar Blue Assay

Cytotoxicity in PBMC and THP-1 cells was evaluated using a resazurin cell viability test. Resazurin sodium salt solution was added to the cells to a final concentration at 0,0125 mg/mL per well. After 2 h at 37 °C, the fluorescence was measured using a Synergy HTX plate reader with λ_ex_ = 530 nm and λ_em_ = 590 nm.

### 2.7 Hemolysis Assays

Blood was obtained from the Central Blood Bank, Lodz, Poland. To isolate the erythrocytes, blood was centrifuged (3000 rpm, 10 min, 4 °C) and washed twice with ice-cold 10 mM PBS (pH = 7.4). The hematocrit was estimated at 14%. Hemolysis was calculated as follows:

$$H\left(\%\right)=\frac{A_{\mathrm{sample}}}{A_{\mathrm{control}}}\times 100\%$$

where *H*(%) is the percent of hemolysis, *A*_sample_ (540 nm) is the absorbance of samples incubated with dendrimers, and *A*_control_ (540 nm) is the absorbance of the positive control.

To evaluate the hemotoxicity of G1–G3 dendrimers, two experiments were conducted. Dendrimers were incubated for 3 h in 5 mM phosphate buffers (pH 4.5, 5.5, and 7.4) and then added to red blood cells mixed with PBS at concentrations in the range of 0.5–25 μM. The negative control consisted of red blood cells incubated with PBS, while for the positive control, cells were exposed to 10% Triton X-100 solution. After 3 h incubation, samples were diluted with PBS to a final hematocrit of 2% and centrifuged (3000 rpm, r.t.). The absorbance at *λ =* 540 nm was measured using a Synergy HTX multimode reader, BioTek. In a second experiment, hemolytic properties of dendrimers were checked in the presence of serum proteins. Samples were prepared as previously described and subsequently incubated for 30 min with human serum at a concentration of 55%.

### 2.8 Interaction with siRNA

Gel electrophoresis was performed on 3% agarose gel in a TAE buffer (1×, Tris-acetate-EDTA) at 90 V and 35 mA for 45 min. The final siRNA concentration was 1 μM. 100 μM dendrimer solutions in H_2_O_DEPC_ were used to prepare the dendrimer/siRNA complexes in a molar ratio of 40:1. Applied pH values were as follows: 4.5, 5.5, and 7.4. Nonmodified and FITC-labeled siRNA (siLuc3) was purchased from Dharmacon (Lafayette, CO) with the sequence as follows: sense, 5′-CUU ACG CUG AGU ACU UCG AdTdT-3′; antisense, 5′-UCG AAG UAC UCA GCG UAA GdTdT-3′. Dendrimer/siRNA complexes were formed in 5 mM phosphate buffer (pH 7.4) by 15 min of incubation at room temperature. After complexation, the pH was adjusted to the desired values using 0.1 M HCl or 0.1 M NaOH H_2_O_DEPC_ solutions. After 30 min of incubation in room temperature, a loading dye was added, and samples were placed onto the wells. The gel was visualized using GelRed nucleic acid staining in a ChemiDoc-It2 camera imager.

### 2.9 Cellular Uptake and Colocalization Analyzed by Confocal Microscopy

To evaluate the internalization of dendrimer/siRNA complexes into A549 cells, a confocal microscopy technique was employed. Confocal imaging was used to visualize the intracellular localization of dendrimer/siRNA complexes and their colocalization with lysosomes. Cells were seeded at a density of 10,000 per well in μ-Slide 18-well glass-bottom plates (Ibidi, Gräfelfing, Germany) and incubated with the respective treatments for 24 h. Subsequently, LysoTracker Red DND-99 (Thermo Fisher Scientific, Waltham, MA) was added at a final concentration of 75 nM and cells were incubated for an additional 2 h at 37 °C to stain the lysosomes. After staining, cells were fixed with 4% paraformaldehyde and counterstained with DAPI (0.5 μg/mL, 5 min) to visualize the nuclei. Fluorescence images were acquired using a Leica TCS SP8 confocal microscope (Leica Microsystems, Wetzlar, Germany) equipped with a 63×/1.40 oil immersion objective (HC PL APO CS2). Excitation/emission settings were as follows: 405/430/470 nm for DAPI, 489/500/530 nm for FITC, and 577/585/610 nm for LysoTracker Red DND-99. Image and colocalization analysis was conducted using Leica LAS X software (v.2.0.215022).

### 2.10 Statistics

Results were presented as the mean with standard deviation. Data normality was estimated with Shapiro–Wilk statistics. The variance homogeneity was verified using the Levene test. The data were analyzed with two-way ANOVA or the Kruskal–Wallis test. The significance of differences between samples was evaluated using Tukey’s posthoc multiple comparisons. All statistical analyses were performed using GraphPad Prism version 10.3.1 for Windows, GraphPad Software, Boston, Massachusetts.

## 3 Results and Discussion

### 3.1 Synthesis and Structural Characterization of Cleavable Carbosilane Dendrimers

In order to overcome one of the main drawbacks of carbosilane dendrimers, that is, their nondegradability, we have recently reported a new family of cleavable dendrimers ArGnV_m_ (I–III).^22^ These dendrimers were employed as cross-linking points in the design of dendritic hydrogels through UV-initiated thiol–ene chemistry (TEC). The presence of cleavable ester bonds at the core as well as multiple aromatic rings in their scaffold was crucial for the behavior of the final materials in drug delivery.

To continue exploring the potential of these cleavable carbosilane dendrimers, we employed dendrimers I–III as precursors of the ammonium-functional dendrimers ArGn­(NMe_2_HCl)_m_ (**1**–**3**), as shown in Figure 1. The synthesis was carried out through the UV-initiated TEC reaction with 2-(dimethylamino)­ethanothiol chloride, in the presence of the photoinitiator DMPA and in a mixture of MeOH:THF:H_2_O (1:0.5:0.5). The products were then isolated after dialysis in methanol and evaporation of the solvent with 70% yield. The reaction was easily monitored by ^1^H NMR through the complete disappearance of vinyl signals and the appearance of multiplets corresponding to the new −S­(CH_2_)_2_NMe_2_HCl fragment at 2.90 and 3.33 ppm and the singlet at 2.90 ppm. As shown in Figure 2 for dendrimer **1**, the aromatic core is identified as a singlet at 8.80 ppm; the triazole rings appear as a singlet at 8.19 ppm, and the closer −OCH_2_– at 5.51 ppm. The methylene groups from the carbosilane region appear in the typical range. In ^13^C NMR spectra, we also confirmed the complete disappearance of vinyl signals and the appearance of those corresponding to the new −S­(CH_2_)_2_NMe_2_HCl fragment at 26.9, 43.7, and 57.9 ppm. The aromatic core is identified at 131.1 and 135.2 ppm, and the triazole rings are at 124.1 and 142.3 ppm, with the closer −OCH_2_– at 59.4 ppm. Again, the methylene groups from the carbosilane scaffold appear in the typical range. All NMR spectra can be found in the Supporting Information (Figure S1–S6).

**Figure 1:**
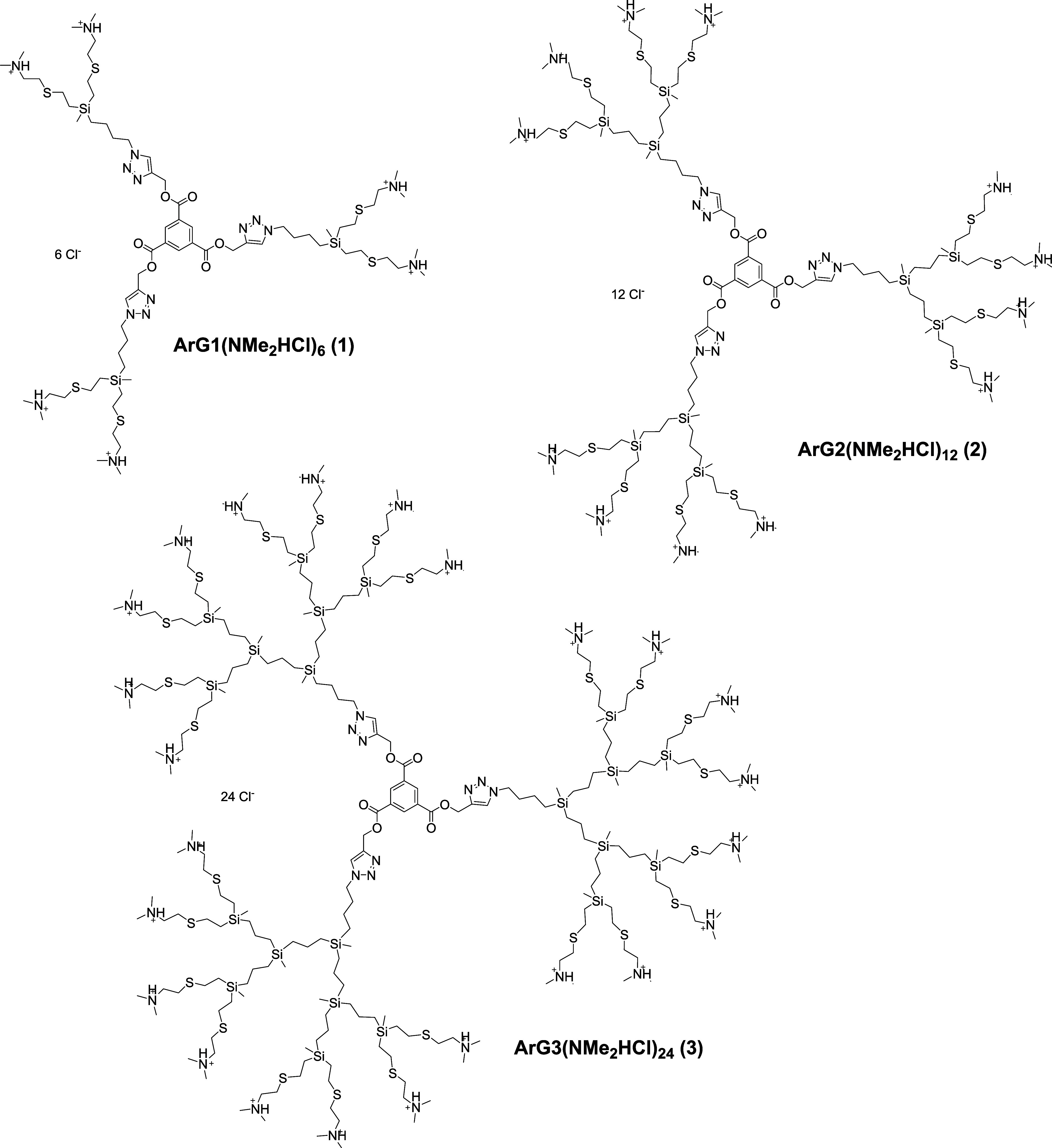
Structures of cleavable cationic dendrimers ArG1­(NMe_2_HCl)_6_ (dendrimer **1**), ArG2­(NMe_2_HCl)_12_ (dendrimer **2**), and ArG3­(NMe_2_HCl)_24_ (dendrimer **3**).

**Figure 2:**
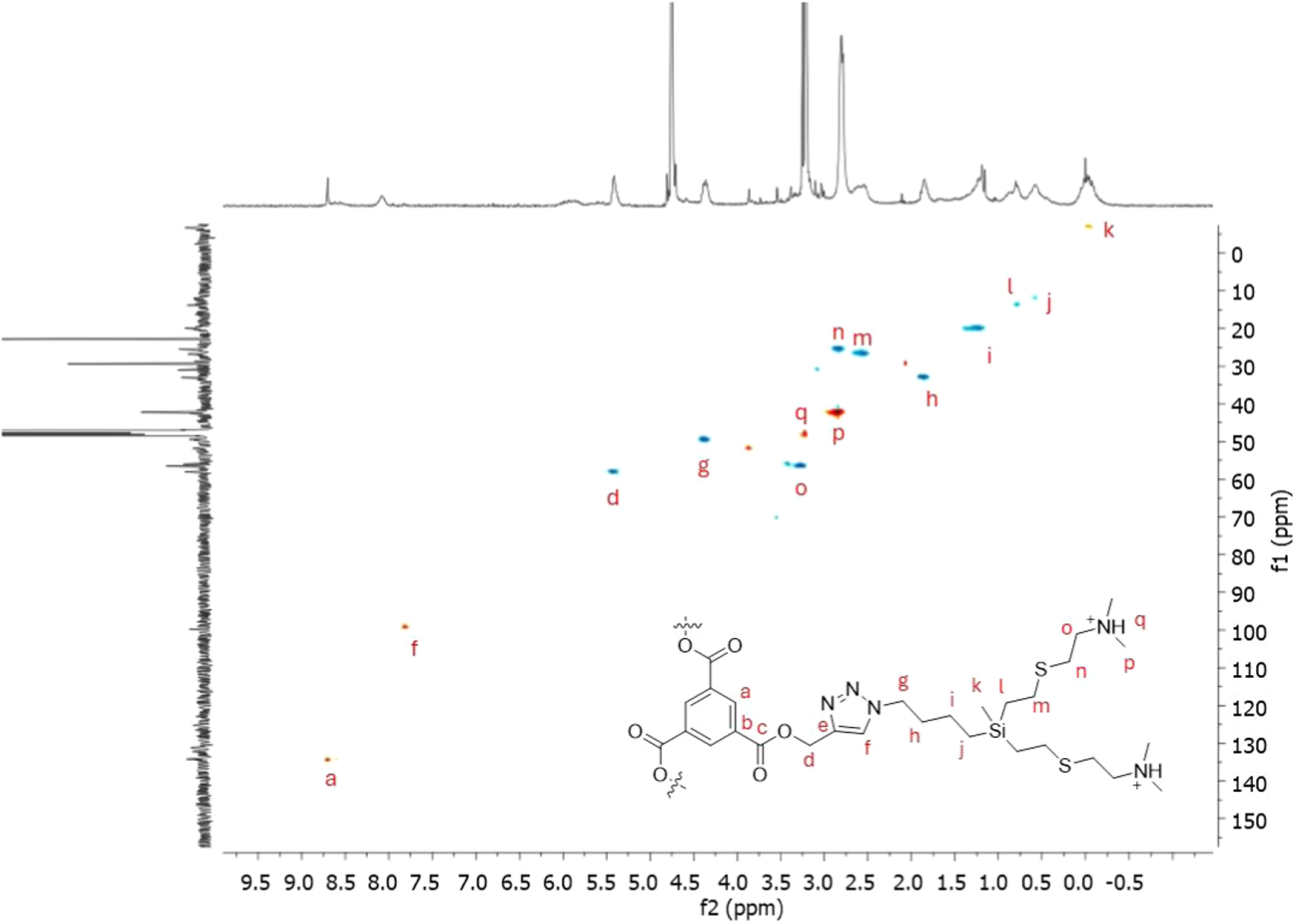
^1^H^13^C-HSQC spectrum in CD_3_OD of cationic dendrimer **1**, with assigned signals.

Dendrimers **1**–**3** were also characterized through elemental analysis and MALDI-TOF. The elemental analysis supported the purity of the compounds. For dendrimer **1**, the molecular peak at 1540.6 Da could be identified by mass spectrometry analysis (Figure S7). For dendrimers **2** and **3**, the molecular peaks could not be identified.

### 3.2 Potentiometric Study

To evaluate the acid–base behavior of the new dendrimers, we performed a potentiometric titration and calculated the p*K*_a_ values using the second derivative method (Figures S8–S10). As summarized in Table 1, dendrimers **1**–**3** exhibit a p*K*_a_ value in the range of 7.9–8.3, assigned to the protonation of the −NMe_2_ peripheral groups. This is a clear difference from many other reported dendrimers with a permanent cationic behavior due to the presence of −NMe_3_^+^ groups. In the present case, the dendrimers will respond to changes in the pH of the medium. Below ∼8.0, the peripheral groups remain as −NMe_2_H^+^, and above it, they will start to be deprotonated. Importantly, a second peak was observed in the curves in the pH range of 4.5–5.2, which could only be assigned to the protonation of the −COO^–^ groups at the core. This indicates that below pH ∼5.0, the ester groups at the core start breaking and the dendrons are cleaved from the core. It is worth highlighting that this cleavage occurs at lower pH values for G2 and G3 dendrimers, confirming a higher tolerance to low pH for higher-generation dendrimers.

**Table 1:** Relevant Parameters for Dendrimers **1**–**3**

dendrimer	*M*_w_ (g/mol)^a^	p*K*_a_ (exp.)	p*K*_a_ (pred.)^c^	size (nm)^d^	zeta potential (mV)^d^
ArG1(NMe_2_HCl)_6_ (**1**)	1547.55	7.9 and 5.2^b^	8.19–9.75	20.0	20.07
ArG2(NMe_2_HCl)_12_ (**2**)	2858.27	8.3 and 4.6^b^	8.07–9.87	5.0	20.60
ArG3(NMe_2_HCl)_24_ (**3**)	5479.71	8.2 and 4.5^b^	8.07–9.87	3.3	22.10

a Calculated with ChemDraw 22.0.0 without considering the Cl^–^ counterions.

b Arising from ester bond rupture.

c Calculated with MarvinSketch 22.7.

d Measured through DLS (average values at 24 h).

Furthermore, we explored the degradation kinetics through ^1^H NMR experiments under different conditions of pH and temperature (Figures S11 and S12). Second-generation dendrimer **2** showed no signs of degradation at pH 7.4 or 5.5 (5 days at 37 °C in each test); however, decreasing the pH at 4.5 revealed an incipient degradation of the molecule. On the contrary, the first-generation counterpart **1** showed a complete cleavage at pH 5.5 (24 h at 37 °C), confirming the dendritic effect observed in the potentiometric titration.

To gain further understanding of the behavior of these polyamine dendrimers, the experimental results were compared with the predicted distribution of macrospecies, determined through MarvinSketch 22.7 (Figures S13–S15). As expected, the titration curves of these dendrimers present a quite complex pattern, considering the increasing number of surface −NMe_2_ groups when moving from G1 (6) to G3 (24). All dendrimers present a set of p*K*_a_ values in the range of 8.1–9.8. This confirms that each nitrogen feels the protonation state of closer nitrogen atoms, as previously described for polyamine macromolecules.^23^ The prediction confirmed that at physiological pH (7.4), the predominant macrospecies present all nitrogen atoms as protonated (Figure S16). A slight increase in the pH starts deprotonating the ammonium groups, which could help release the cargo, and below pH 5.8, the predominant macrospecies have all −NHMe_2_^+^ groups. According to the prediction, at physiological pH, around 15% of molecules have one deprotonated group, and on increasing the pH to 8.0, half of the molecules have all protonated groups or with 1–2 −NMe_2_ groups.

These experiments confirmed that the newly synthesized dendrimers are cleavable under acid hydrolysis and potentially under enzymatic action and present pH-responsive properties.

### 3.3 Biophysical Characterization of Cleavable Dendrimers

The hydrodynamic diameter and zeta potential of the dendrimers were studied through DLS assays. Samples were dissolved in distilled water and measured in 5 mM sodium phosphate buffer at a final concentration of 20 μM. The pH was adjusted to 7.4, 5.5, and 4.5 to evaluate the impact of the dendrimers’ protonation and/or cleavage. Changes in the size and zeta potential were registered for 24 h. Results are summarized in Figure 3.

**Figure 3:**
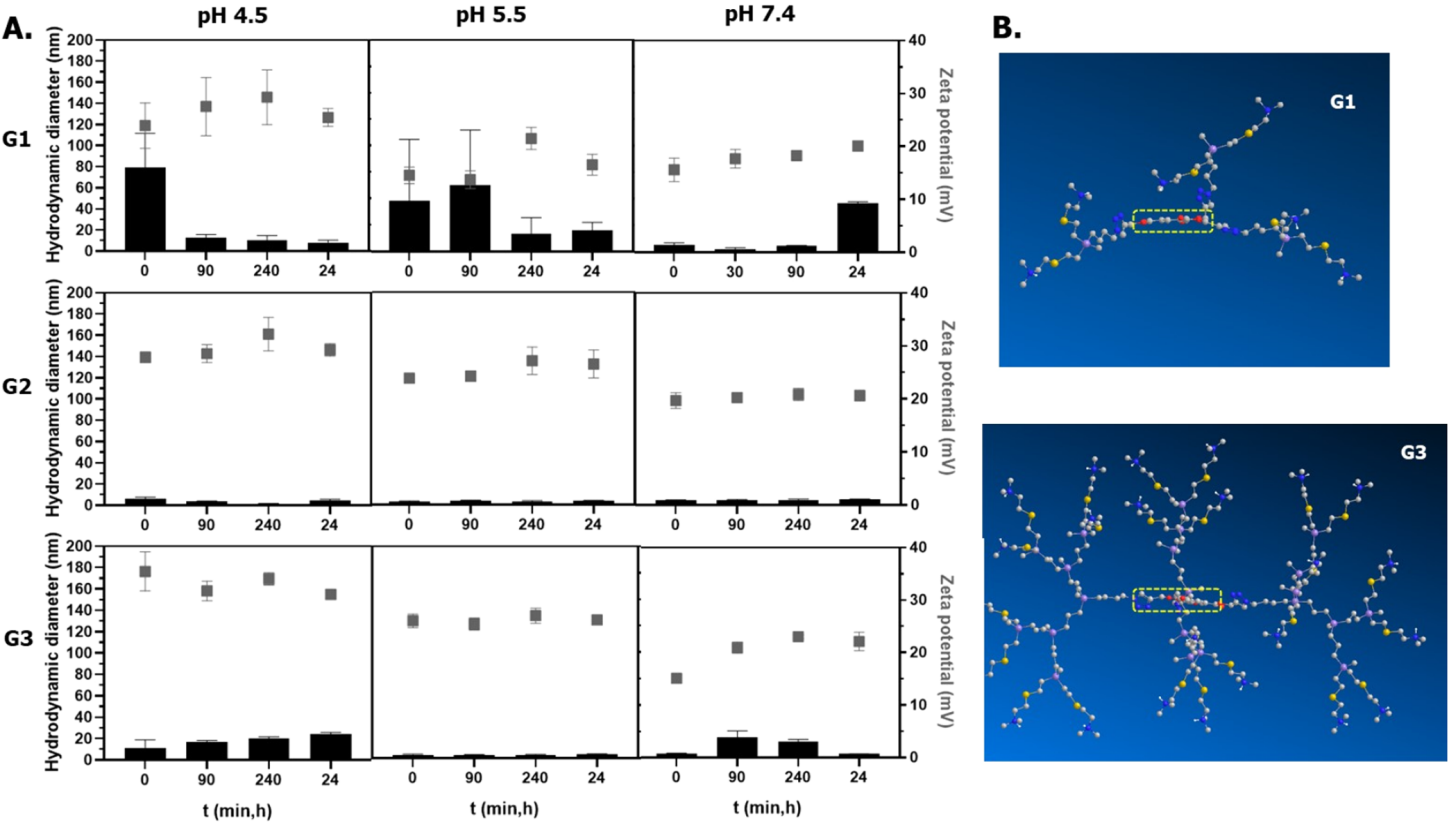
(A) Hydrodynamic diameter (left axis, bars) and zeta potential (right axis, squares) values for cleavable dendrimers **1**–**3** at pH values of 4.5, 5.5, and 7.4. (B) Snapshots from the 3D spatial arrangement of carbosilane dendrimers **1** and **3** after MD jobs. The dendrimer core is highlighted [Chem3D software: Job 1 (minimize energy to a minimum RMS gradient of 0.010) + Job 2 (MD; step interval: 2.0 fs; frame interval: 10 fs; terminate after: 10,000 steps; heating/cooling rate: 1.000 kcal/atom/ps; target temperature: 300 K)].

As expected, zeta potential values were positive, in line with the cationic behavior of the dendrimers, and they progressively decreased from pH 4.5 to 7.4. In general, within each experiment, the values remained approximately constant, confirming the stability of the dendrimers in solution over time.

Regarding the hydrodynamic diameter, relevant differences were observed between the higher-generation systems (G2 and G3) and the smaller counterpart (G1).

At pH 5.5, when all nitrogen atoms are protonated, G2 and G3 dendrimers exhibit average sizes around 3 nm and zeta potential values around 23 mV, which is in line with the strong cationic charge. This produces strong repulsion between the different particles, thus preventing their aggregation and improving their colloidal stability. A different behavior was found for G1, with an average size around 20 nm after 24 h and lower zeta potential values around 15 mV, indicating lower colloidal stability in solution. This is also in agreement with the results obtained from PdI analysis (Figure S17). For G1, PdI values increase over time, especially at pH 4.5 and 5.5. On the contrary, for G2 and G3 dendrimers, PdI values substantially decrease over time, mainly at pH 5.5 and 7.4.

To gain further insight into this behavior, molecular dynamic simulations were run for dendrimers **1**–**3** at the full protonation state (Figure 3B). We observed a very open conformation for the G1 dendrimer, which could establish intermolecular interactions through the trimesic-core ring stacking. It is known that trimesic acid (TMA) forms strong intermolecular interactions, through H bonding and ring stacking.^24^ On the other hand, in G2 and G3 counterparts, the steric hindrance and charge repulsion posed by the positively charged branches minimize access to the core and thus interdendrimer interactions. This may be the reason the size remains around 3 nm over the span of the experiment.

If the pH is increased to physiological values (7.4), the predominant macrospecies presents all nitrogen atoms as protonated, but some nitrogen atoms start being deprotonated. This is confirmed by a slight decrease of the zeta potential value (20 mV) and a slight increase in size (5.0 and 3.3 nm) for G2 and G3, respectively. For G1, with average values of around 46.8 nm after 24 h, the increase in pH seems to produce a less favorable environment for intermolecular interactions. On the contrary, if the pH is decreased to 4.5, when the dendrimers are fully protonated but start being cleaved, we did not observe statistically significant changes over time. In general, the aggregate formation should be promoted due to easier accessibility to the TM core when some branches are detached.

### 3.4 Biocompatibility of Cleavable Dendrimers

The biocompatibility of dendrimers **1**–**3** was evaluated in kidney HEK-293 cells as well as in nontumoral monocytes (PBMCs) and leukemia monocytes (THP-1) in the concentration range of 0.1–10 μM. Dendrimer solutions were prepared in 5 mM phosphate buffer at different pH values and incubated for 3 h at r.t. before cell treatment. Selected pH values were 5.5 when dendrimers are fully protonated, 7.4 when deprotonation starts occurring and a small amount of −NMe_2_ groups may be present, and 4.5 to evaluate the effects of already cleaved dendrimers. The results are summarized in Figure 4.

**Figure 4:**
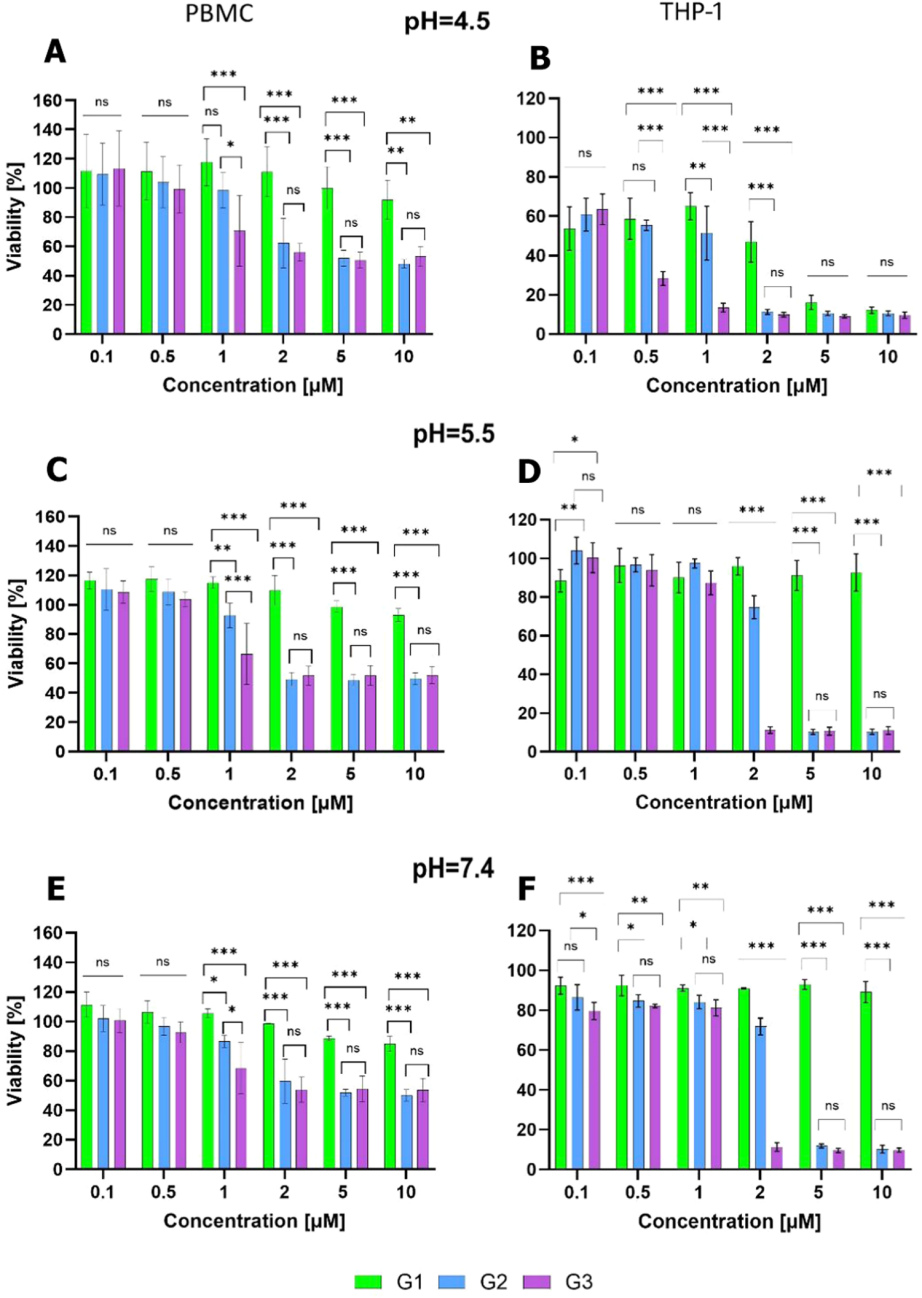
PBMC and THP-1 cell viability after 24 h incubation with G1–G3 dendrimers at pH (A, B) 4.5, (C, D) 5.5, and (E, F) 7.4. Left: nontumoral PBMC. Right: tumoral THP-1. Results presented as mean ± SD, *n* = 3, *p ≤ 0.05, **p ≤ 0.01, ***p ≤ 0.001.

As expected, cell toxicity depended on dendrimer generation and concentration, as well as on the preincubation pH. In PBMC and THP-1 cells, similar trends were observed. G1 dendrimer **1** was biocompatible in all concentration ranges at both pH 5.5 and 7.4. A slightly higher toxicity was observed at pH 7.4, when deprotonation starts to occur and a small percentage of −NMe_2_ is present.

This fact may be related to a slight change in amphiphilicity, with the dendrimer turning a bit more lipophilic. The cytotoxicity increased with the dendrimer generation. Dendrimers **2** and **3** were biocompatible in PBMCs up to 1 and 0.5 μM, respectively, and slightly higher concentrations in THP-1 (1 μM). It is worth noting that at pH 4.5, all cleaved dendrimers exhibited relevant toxicity in THP-1 cancer cells even at the lowest concentrations. This did not occur in PBMCs and could indicate a potential selective effect on cancer cells. The pattern observed in monocytes is also observed in HEK-293 cells, but with a slightly higher toxicity in the latter (Figure S18).

### 3.5 Hemolysis

The cytotoxic effect on erythrocytes was studied for different dendrimers, in the concentration range of 0.1–25 μM. Dendrimer solutions were prepared at different pH values, like in the previous biocompatibility assays. The results are presented in Figure 5. The first-generation dendrimer **1** showed low hemotoxicity (below 20%) up to 10 μM at pH 5.5, and it was slightly higher at pH 7.4 due to slight deprotonation. For the second- and third-generation counterparts, only dendrimer **2** at pH 5.5 was below the 20% hemolysis limit. In general, the hemotoxicity increased with the incubation pH, with the cleaved dendrimers at pH 4.5 showing less toxicity toward erythrocytes.

**Figure 5:**
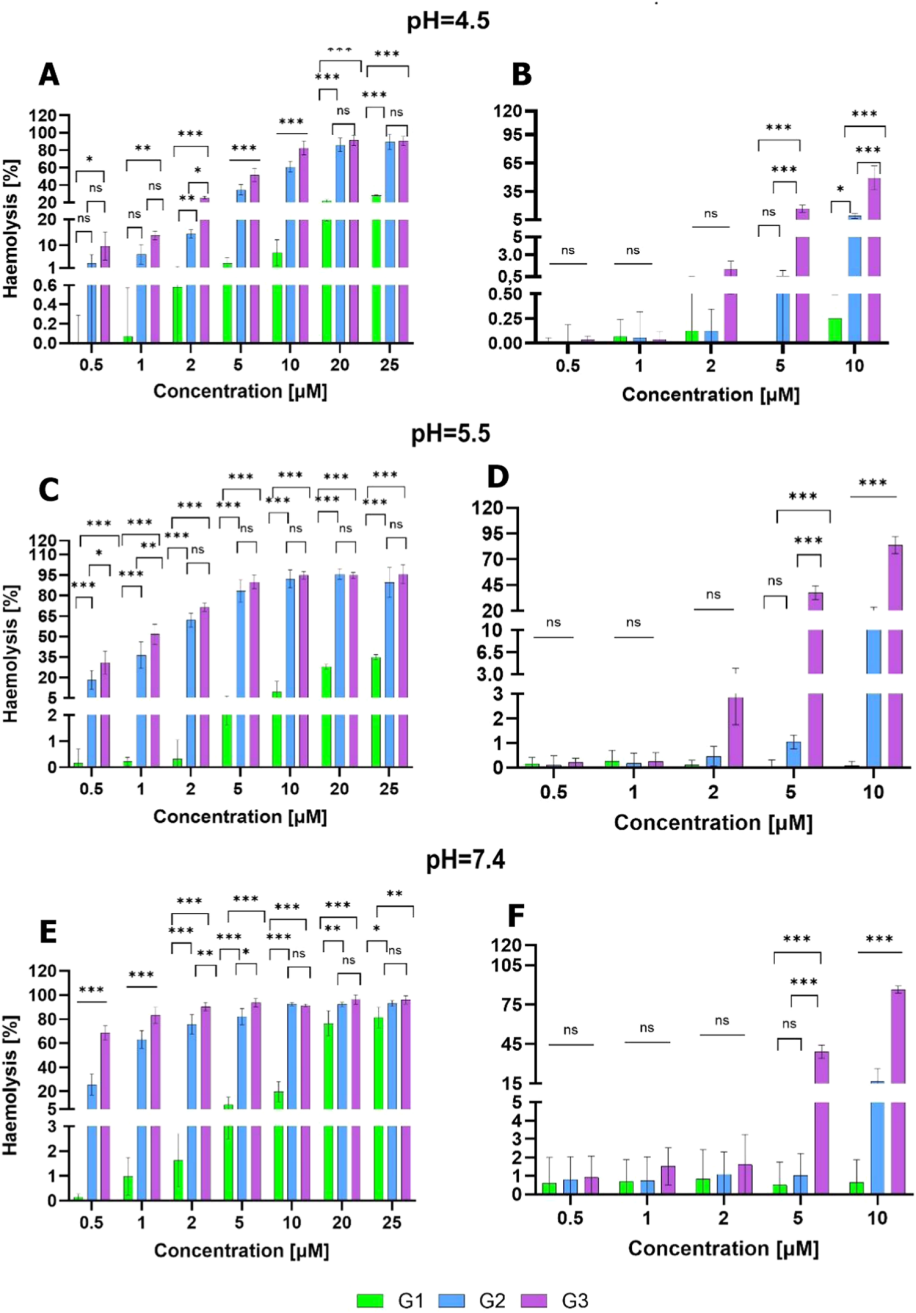
Hemolysis effect of G1–G3 dendrimers at pH (A, B) 4.5, (C, D) 5.5, and (E, F) 7.4, alone (left) and after incubation with 55% serum (right). Results presented as mean ± SD, *n* = 3, *p ≤ 0.05, **p ≤ 0.01, ***p ≤ 0.001.

As can be seen in Figure 5B–F, additional incubation with serum proteins resulted in a significant decrease in dendrimer-induced hemolysis. Hemotoxicity induced by the G1 dendrimer was nonsignificant in all pH levels and in all tested concentrations. The G2 dendrimer was also below the 20% hemolysis limit at all concentrations tested, while the G3 dendrimer caused hemolysis at concentrations higher than 5 μM. We can conclude that the compounds presented herein follow a similar pattern to previous carbosilane dendrimers; the dendrimers’ cytotoxicity correlated with their generation and decreased after their interaction with serum proteins.^25^

### 3.6 Interaction with siRNA and Cell Uptake

Considering the higher stability of G2 and G3 dendrimers in solution as well as the higher number of cationic charges (12 and 24 positive charges, respectively) which can strongly bind nucleic acids, the interaction with siRNA was explored through agarose gel electropherograms compared to the G1 dendrimer counterpart. Samples containing 1 μM siRNA and dendrimers at different molar ratios were prepared in a 5 mM sodium phosphate buffer (pH 7.4). Complexes were incubated for 15 min at room temperature before the electrophoresis. As depicted in Figure 6A, G2 and G3 dendrimers efficiently generated dendriplexes with siRNA. At a dendrimer:siRNA ratio of 5:1, the interaction becomes strong enough to start retaining some siRNA in the well, but it is much more intense for dendrimer **3**. For the first-generation dendrimer **1**, a much higher ratio is required to fully complex the siRNA.

**Figure 6:**
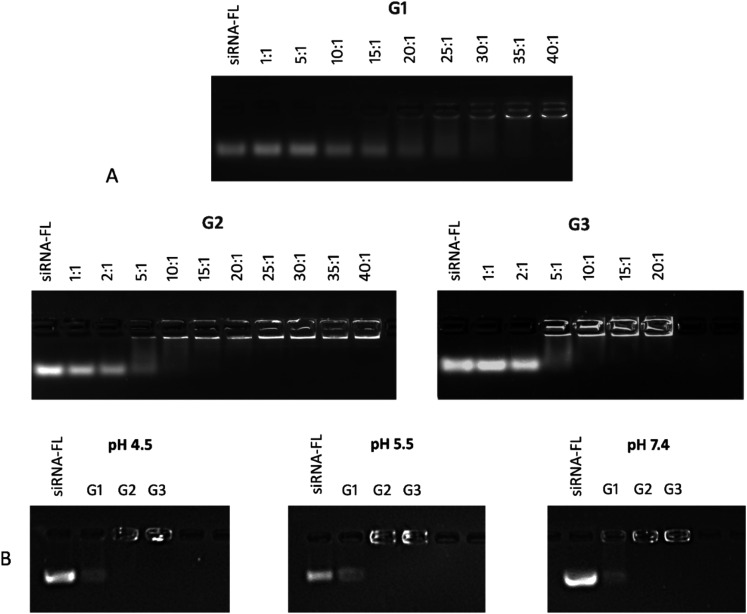
Agarose gel electropherograms of siRNA complexed with dendrimers G1, G2, and G3. (A) Complexation study at increasing dendrimer concentrations. Concentration of siRNA: 1 μmol/L, pH: 7.4. (B) siRNA release from dendrimer/siRNA complexes at a molar ratio of 40:1 at pH = 4.5, 5.5, and 7.4. Line 1 demonstrates the migration of naked siRNA. Lines 2–4 demonstrate the migration of dendrimer/siRNA complexes.

A second experiment was performed to evaluate the effect of the pH at the dendriplex formation stage. In this case, dendrimer/siRNA complexes (ratio 40:1) were formed at pH 7.4 for 15 min at r.t.; subsequently, the pH was adjusted to pH 4.5 or 5.5, and they were incubated for an additional 30 min at r.t. Under these conditions, G2 and G3 dendrimers retained the siRNA at all tested pH values; however, some siRNA release was observed for the G1 dendrimer, especially at a lower pH, due to its lower buffering capacity or initial ester cleavage (Figure 6B).

Measurements of the hydrodynamic diameter and zeta potential of siRNA complexed with dendrimers were performed by DLS and zeta potential techniques (Figure 7). The zeta potential of the formed siRNA/dendrimer complexes switched from negative to positive values with the changing dendrimer:siRNA molar ratio. This value depended on the dendrimer generation: 50:1 (G1), 30:1 (G2), and 10:1 (G3), in line with the increase in the number of −NHMe_2_^+^ groups. Regarding the hydrodynamic diameter, at the same dendrimer/siRNA molar ratio of 40:1, G1 generated complexes around 450 nm, while the G2 and G3 counterparts produced bigger complexes around 510–525 nm (Figure 7). DLS measurements of unbound siRNA revealed comparatively large particle sizes, which can be attributed to the formation of high-molecular-weight aggregates by uncomplexed siRNA molecules. This aggregation is disfavored in the presence of the cationic dendrimers, which interact with siRNA.

**Figure 7:**
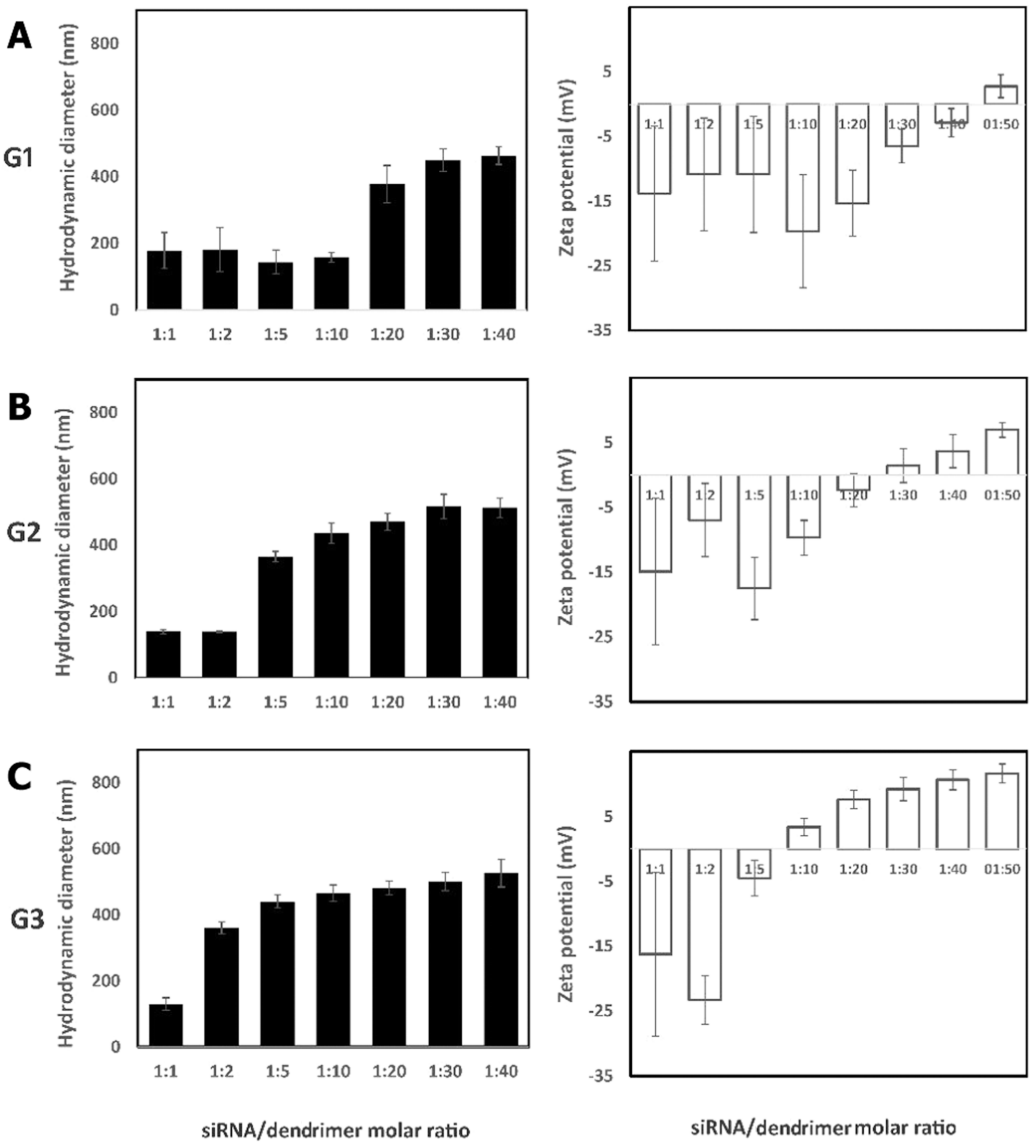
Hydrodynamic diameters (left panels) and zeta potentials (right panels) of siRNA/dendrimer complexes in increased molar ratios for (A) G1, (B) G2, and (C) G3 dendrimers. siRNA concentration: 0.3 μM. The results represent mean values with standard deviations (*n* = 3).

To confirm that the cleavable dendrimers are potential siRNA carriers *in vitro*, human lung adenocarcinoma A549 cells were incubated with the dendriplexes for 24 h, and subsequently confocal imaging was used to visualize their intracellular localization. Fluorescence signals corresponding to siRNA-FITC could be clearly detected in all groups treated with dendrimer/siRNA complexes (Figure 8). In contrast, the cells exposed to noncomplexed siRNA-FITC showed a minimal signal. Only 8% uptake was observed for naked siRNA after 24 h; however, the complexation with the dendrimers significantly increased the uptake to 50% (G1) and 32–35% (G2 and G3). Probably, the smaller size of the siRNA/G1 complex facilitated the cell uptake. Additionally, to investigate intracellular distribution of the complexes, the colocalization with Lysotracker Red was assessed. Quantitative image analysis indicated that the G1 dendrimer exhibited the highest colocalization rate (74.8  ±  10.0%), suggesting that a substantial proportion of the delivered siRNA remained within lysosomal compartments. These findings suggest that while G1 can provide efficient cellular uptake, it is also associated with pronounced lysosomal accumulation. In contrast, G3 offers a favorable balance between uptake and cytoplasmic availability, making it a promising candidate for functional siRNA delivery.

**Figure 8:**
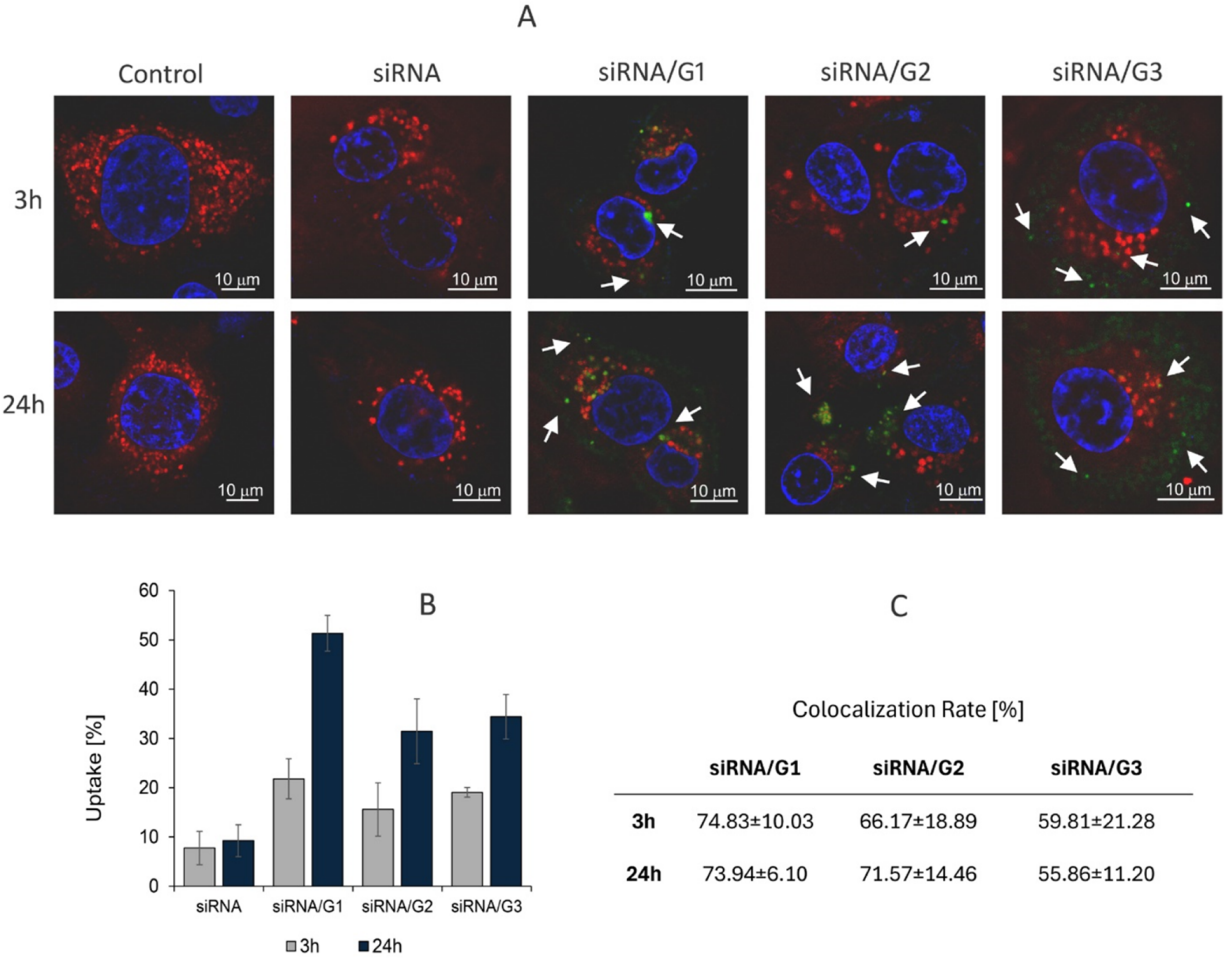
Intracellular distribution and lysosomal colocalization of siRNA/dendrimer complexes analyzed by confocal microscopy. (A) Confocal microscopy images at 3 and 24 h. (B) Uptake (%) of FITC-labeled siRNA (siLuc3) by A549 cells by the different dendriplexes, compared to naked siRNA. (C) Quantitative colocalization analysis based on Pearson’s correlation coefficient and colocalization rate (%), indicating differences in lysosomal entrapment among the tested samples. Complexes were performed in PBS 10 mmol/L, pH 7.4 with an incubation time of 24 h. The concentration of siRNA was 100 nM. Control: untreated cells. Cell nuclei are stained with DAPI (blue), lysosomes are stained with Lysotracker Red (red), and siLuc3-FITC is visualized in green. Colocalization appears in yellow in the merged channels. Results are represented as mean ± SD, *n* = 6.

## 4 Conclusions

Carbosilane dendrimers containing C–Si bonds, in contrast to those possessing O–Si bonds, exhibit high stability in aqueous solutions.^26^ The cleavable dendrimers herein presented appear as a relevant step forward, as their controlled fragmentation in smaller dendritic fragments can be beneficial in biomedical applications, that is, easier body clearance. The cleavage of the dendritic core occurs at low pH: below 5 for G1 or below 4.5 for G2 and G3. This confirms the impact of the dendrimer generation on the stability to pH. The cleavage could also be assisted by esterases, as we have previously shown.^22^

The pH can also modulate the protonation state of the peripheral −NMe_2_ groups. At pH 5.5, all groups are protonated; however, at pH 7.4, a small percentage is deprotonated. This can modulate the interaction with negatively charged siRNA. Other pH-responsive dendrimers have been reported in the literature. For example, Shen et al. reported the first dual pH- and temperature-responsive dendrimers, based on poly­(b-aminoester) scaffolds.^27^ The authors confirmed that their sensitivity to pH and temperature is associated with the protonation–deprotonation of peripheral tertiary amine groups. The G4 dendrimers showed two p*K*_a_ values: 7.0 ascribed to the surface amine groups and 2.3 ascribed to the interior tertiary amine groups.

The behavior of dendrimers depends not only on their generation but also on the presence of the trimesic core. For the vinyl-functional counterparts, MD studies showed a planar arrangement of the dendritic core, which included the phenyl ring and the ester bonds and a cagelike orientation of the carbosilane branches in the opposite direction, facilitating the exposure of the core.^22^ After modification with −NMe_2_H^+^ groups, the cationic repulsion generates a more uniform arrangement of the branches in all directions. Anionic carbosilane dendrimers, exhibiting either the silicon or polyphenoxo core, confirmed the major impact of the core architecture.^28^ Unlike the open structure observed when simulated in vacuum, in a water environment, they are closely packed except for peripheral charged residues. Furthermore, the protonation of terminal amines modulates the eventual backfolding of dendrimer branches toward the core. A similar situation could happen for the trimesic-core dendrimers herein reported.

It is worth noting that these cleavable carbosilane dendrimers with −NMe_2_ groups are readily water-soluble, which enables their use in biomedical applications. Most of the −NMe_2_ decorated carbosilane counterparts, e.g., with the silicon^14^ or GnO3 core,^29^ require their conversion to −NMe_3_^+^ to be dissolved, as in the −NMe_2_ form, they tend to aggregate upon exposure to water and hinder their dissolution. Besides water solubility, for a gene therapy application, the use of biocompatible vectors is crucial. The cleavable carbosilane dendrimers herein described showed cell toxicity depending on dendrimer generation and concentration, as well as on the preincubation pH. In PBMC and THP-1, G1 was biocompatible up to 10 μM; however, this was lower for G2 and G3 (∼1 μM) due to the higher number of cationic groups. A surprisingly high toxicity was observed for all cleaved dendrimers at pH 4.5 in THP-1 cancer cells, which did not occur in PBMCs, and this could indicate a potential selective effect against cancer cells. Compared with other cationic carbosilane dendrimers, similar results were found. For example, GnO_3_-cored dendrimers with −NMe_3_^+^ (with 6, 12, and 24 groups) were biocompatible in PBMCs up to 1 μM.^27^ However, their complexation with nucleic acids led to a drastic reduction of cytotoxicity. Regarding hemotoxicity, at 1 μM, all dendrimers were highly hemotoxic (G1, 35%; G2, 48%; G3, 87%), but in whole blood, they were safe up to 3–5 μM. A similar behavior is found for the cleavable dendrimers, but in the presence of blood proteins, G1 and G2 are biocompatible up to 10 μM. The silicon-cored G2 dendrimer decorated with 8 −NH_3_^+^ groups was cytotoxic in PBMCs above 1 μM, while a similar Janus dendrimer with 8 −NH_3_^+^ groups and PEG chains was biocompatible up to 10 μM,^17^ showcasing the impact of the linear chains. Regarding the difference in sensitivity for PBMC and endothelial HEK-293 cells, it is probably related to the high metabolic activity and growth speed of the latter.

As previously reported, and also demonstrated in this work, the use of ionizable dendrimers as nonviral vectors for RNA delivery is a promising strategy.^30^ At physiological conditions, they present enough positive charge to bind anionic RNA, while the surface potential of the formed complex remains equilibrated to promote cellular uptake without being cleared by the immune system. In this sense, ionizable carbosilane dendrimers demonstrated their potential and join the library of amphiphilic dendrimers for RNA delivery, which mainly includes Janus,^30^ core–shell,^31^ and dendron-tail systems.^32^ Siegwart et al. employed a systematic approach to design degradable dendrimers as miRNA carriers.^33^ From the library of more than 1500 candidates prepared, with diversified cores, peripheries, and generations, they found that dendrimers with an siRNA-binding core and an NP-stabilizing periphery had a much higher intracellular siRNA delivery potential. In our case, the presence of the cleavable trimesic-ester core and the hydrophobic scaffold seemed crucial for their use as siRNA carriers. Peng et al. have broadly explored the design of ionizable dendritic micelles formed by amphiphilic PAMAM dendron-tail systems, which formed stable complexes with siRNA with 40 nm and a surface potential of +18 mV. They confirmed the effective internalization of siRNA within Panc-1 cancer cells via endocytosis and endosomal escape after 5 h of incubation, as they disassemble readily under acidic conditions. Furthermore, this supramolecular system is highly dependent on the concentration in the medium, with CMC 19 μM at pH 7.4. The ionizable carbosilane dendrimers offer high stability, not dependent on the concentration, and a prolonged tolerance to acidic media. The complexes with siRNA exhibited a lower surface potential, with enough colloidal stability to remain in solution but beneficial to promote cellular uptake and the possibility to modulate endosomal escape through dendrimer generation.

Overall, core-cleavable carbosilane dendrimers appear as promising new vectors for the delivery of siRNA. Their hydrodynamic diameter and zeta potential respond to changes in pH, but the higher generations (G2 and G3) form strong complexes with siRNA even at low and high pH values, protecting the cargo from degradation. Ongoing experiments will confirm their potential in preclinical assays.

## Supplementary Material



## Notes

Characterization of dendrimers **1**–**3**; ^1^H, ^13^C, and HSQC NMR spectra; MALDI-TOF; potentiometric titration curves; prediction of macrospecies distribution; and toxicity assays in HEK-293 cells (PDF)
