# Supplementary material for: Cleavable Cationic Carbosilane Dendrimers with pH-Tunable Charge as siRNA Carriers
Source: Biomacromolecules. 2025 Jul 18;26(8):4910–23. doi: 10.1021/acs.biomac.5c00344 (PMC12344721; doi:10.1021/acs.biomac.5c00344)
Supplement: Supplementary file 1 [file bm5c00344_si_001.pdf]

Electronic Supporting Information

Cleavable cationic carbosilane dendrimers with pH-tunable charge as siRNA carriers

Judith Recio-Ruiz,[a] Paulina Rycharska,[b,c] Małgorzata Grygiel,[b] Sylwia Michlewska,[d] Maria Bryszewska,[b] Maksim Ionov, [b,e] Francisco Javier de la Mata,[a,f,g] and Sandra García-Gallego, [a,f,g]*

a. University of Alcala, Department of Organic and Inorganic Chemistry and Research Institute in Chemistry "Andrés M. Del Río" (IQAR), 28805, Madrid, Spain. judith.recio@edu.uah.es

b. University of Lodz, Faculty of Biology and Environmental Protection, Department of General Biophysics, Pomorska 141/143, 90-236 Lodz, Poland.

c. BioMedChem Doctoral School of the University of Lodz and Lodz Institutes of the Polish Academy of Sciences. Matejki 21/23, 90-237 Lodz, Poland.

d. University of Lodz, Faculty of Biology and Environmental Protection, Laboratory of Microscopic Imaging and Specialized Biological Techniques, Banacha 12/16, 90-237 Lodz, Poland.

e. Faculty of Medicine, Collegium Medicum, Mazovian Academy in Plock, Pl. Dabrowskiego 2, 09-402 Plock, Poland.

f. Networking Research Center on Bioengineering, Biomaterials and Nanomedicine (CIBER-BBN), 28029, Madrid, Spain;

g. Institute Ramón y Cajal for Health Research (IRYCIS), 28034, Madrid, Spain.

Table of contents

Scheme S1. Synthesis of dendrimer **ArG1(NMe$_2$HCl)$_6$ (1)**. 3

Scheme S2. Synthesis of dendrimer **ArG2(NMe$_2$HCl)$_{12}$ (2)**. 3

Scheme S3. Synthesis of dendrimer **ArG3(NMe$_2$HCl)$_{24}$ (3)**. 4

Figure S1. ^{1}H and ^{13}C NMR spectra of dendrimer **(1)** in MeOD. 5

Figure S2. ^{1}H-^{13}C HSQC spectrum of dendrimer **(1)** in MeOD. 6

Figure S3. ^{1}H and ^{13}C NMR spectra of dendrimer **(2)** in MeOD. 7

Figure S4. ^{1}H-^{13}C HSQC spectrum of dendrimer **(2)** in MeOD. 8

Figure S5. ^{1}H and ^{13}C NMR spectra of dendrimer **(3)** in MeOD. 9

Figure S6. ^{1}H-^{13}C HSQC spectrum of dendrimer **(3)** in MeOD. 10

Figure S7. MALDI-TOF spectra of dendrimer **(1)** in DHB matrix. 10

Figure S8. Potentiometric study of dendrimer **(1)** in water. 11

Figure S9. Potentiometric study of dendrimer **(2)** in water. 11

Figure S10. Potentiometric study of dendrimer **(3)** in water. 11

Figure S11. Degradation study of dendrimer **(2)** at different pH. 12

Figure S12. Degradation study of dendrimer **(1)** at different pH. 12

Figure S13. Macrospecies calculation of dendrimer **(1)** with MarvinSketch 22.7. 13

Figure S14. Macrospecies calculation of dendrimer **(2)** with MarvinSketch 22.7. 13

Figure S15. Macrospecies calculation of dendrimer **(3)** with MarvinSketch 22.7. 14

Figure S16. Comparative macrospecies distribution (%) at selected pH values (2.8, 4.2, 5.8, 7.4, 8.0 and 11.2) for dendrimers **1-3**. 15

Figure S17. Evolution of PDI over time for dendrimers **1-3** at different pH. 16

Figure S18. HEK-293 cell viability after 24h incubation with G1-G3 dendrimers at different pH. 16

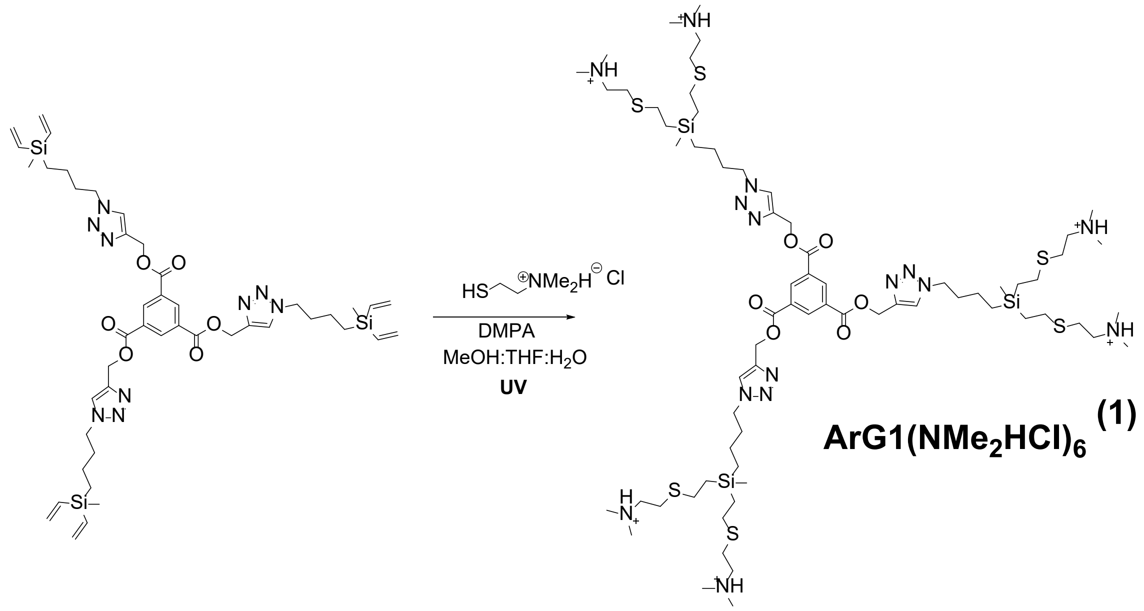

Scheme S1. Synthesis of dendrimer **ArG1(NMe$_2$HCl)$_6$ (1)**.

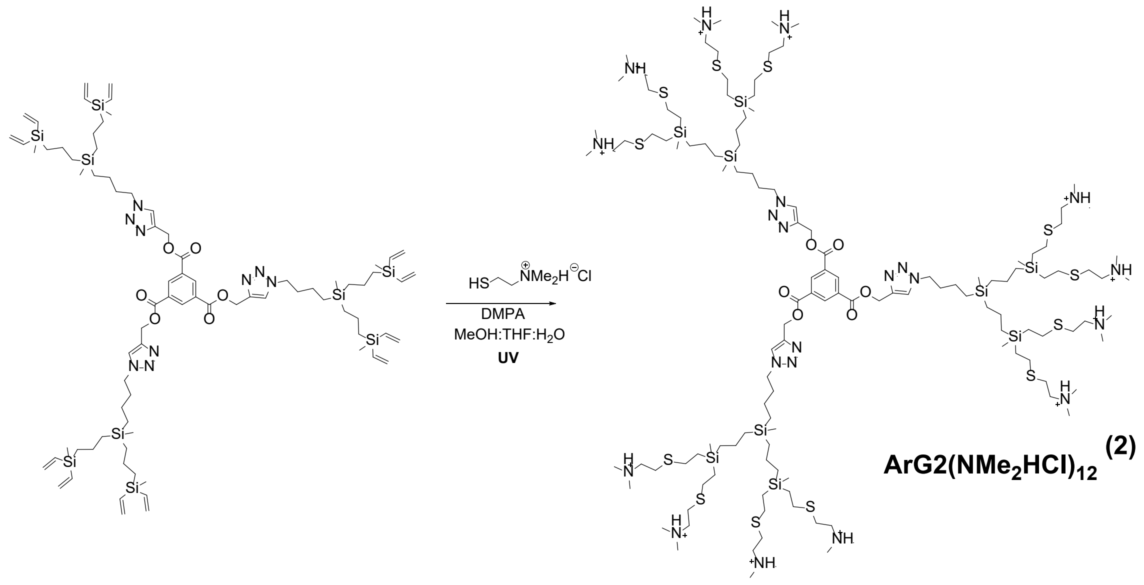

Scheme S2. Synthesis of dendrimer **ArG2(NMe$_2$HCl)$_{12}$ (2)**.

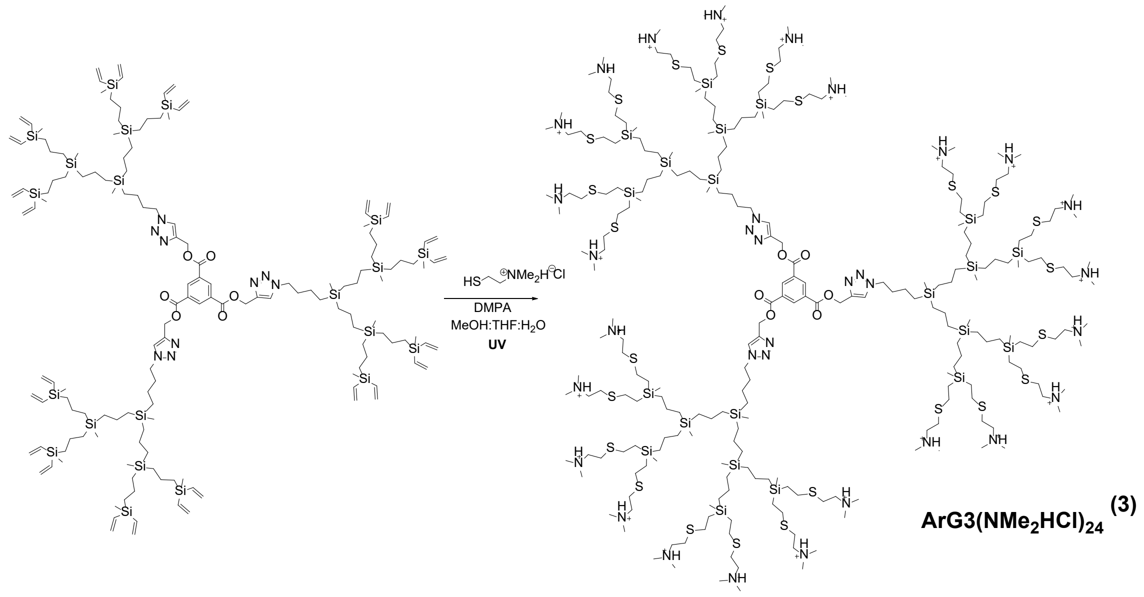

Scheme S3. Synthesis of dendrimer **ArG3(NMe$_2$HCl)$_{24}$ (3)**.

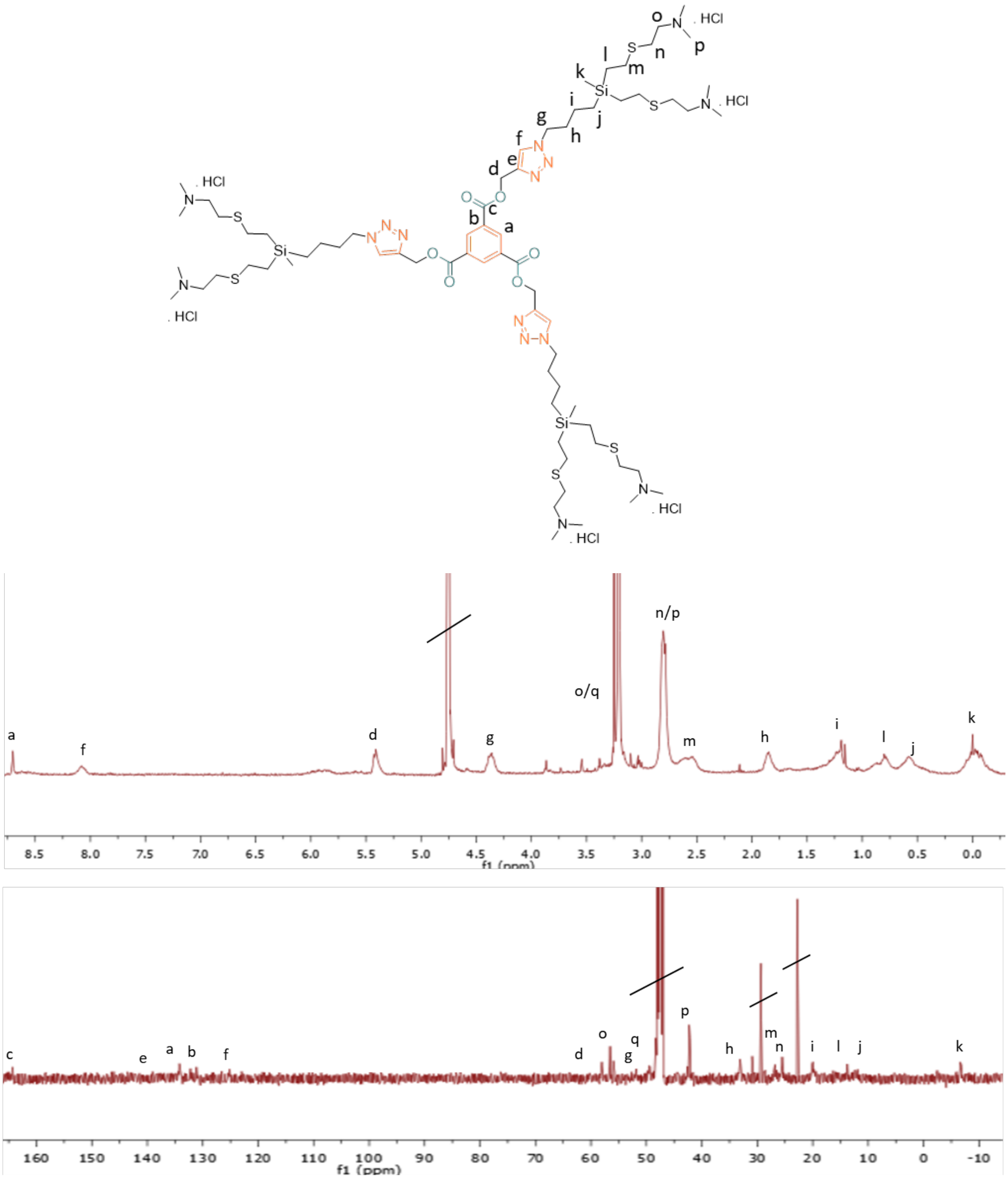

Figure S1. ^{1}H and ^{13}C NMR spectra of dendrimer **(1)** in MeOD.

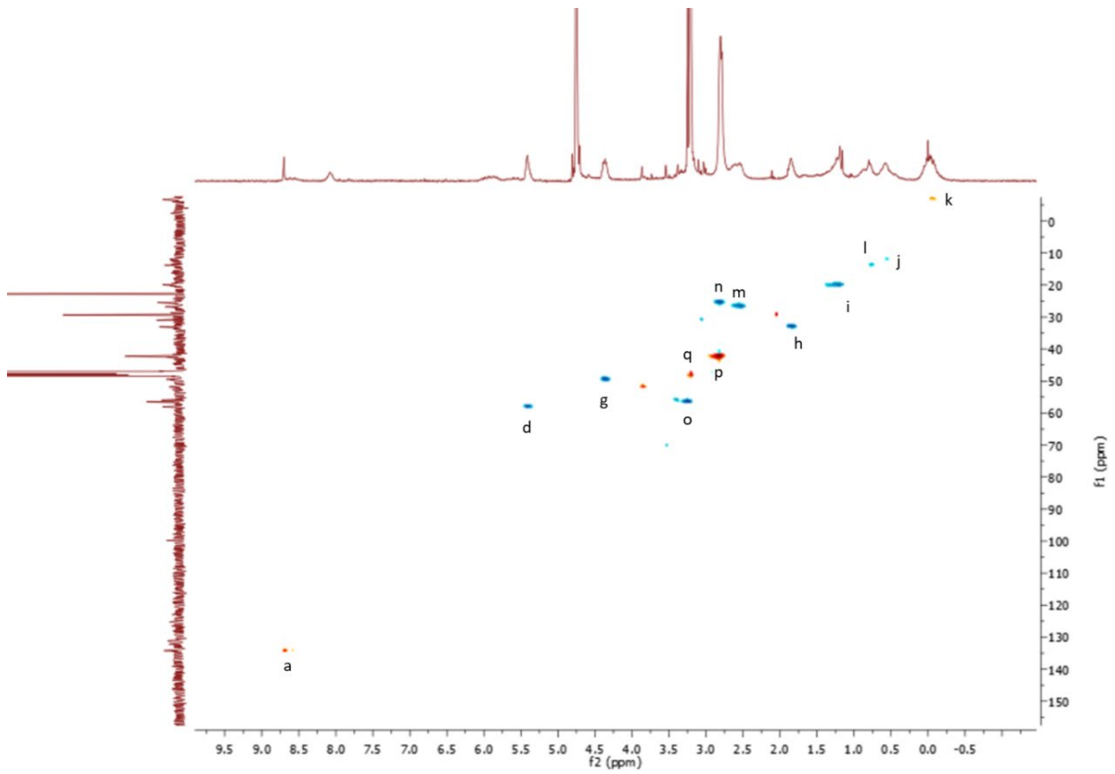

Figure S2. ^{1}H-^{13}C HSQC spectrum of dendrimer **(1)** in MeOD.

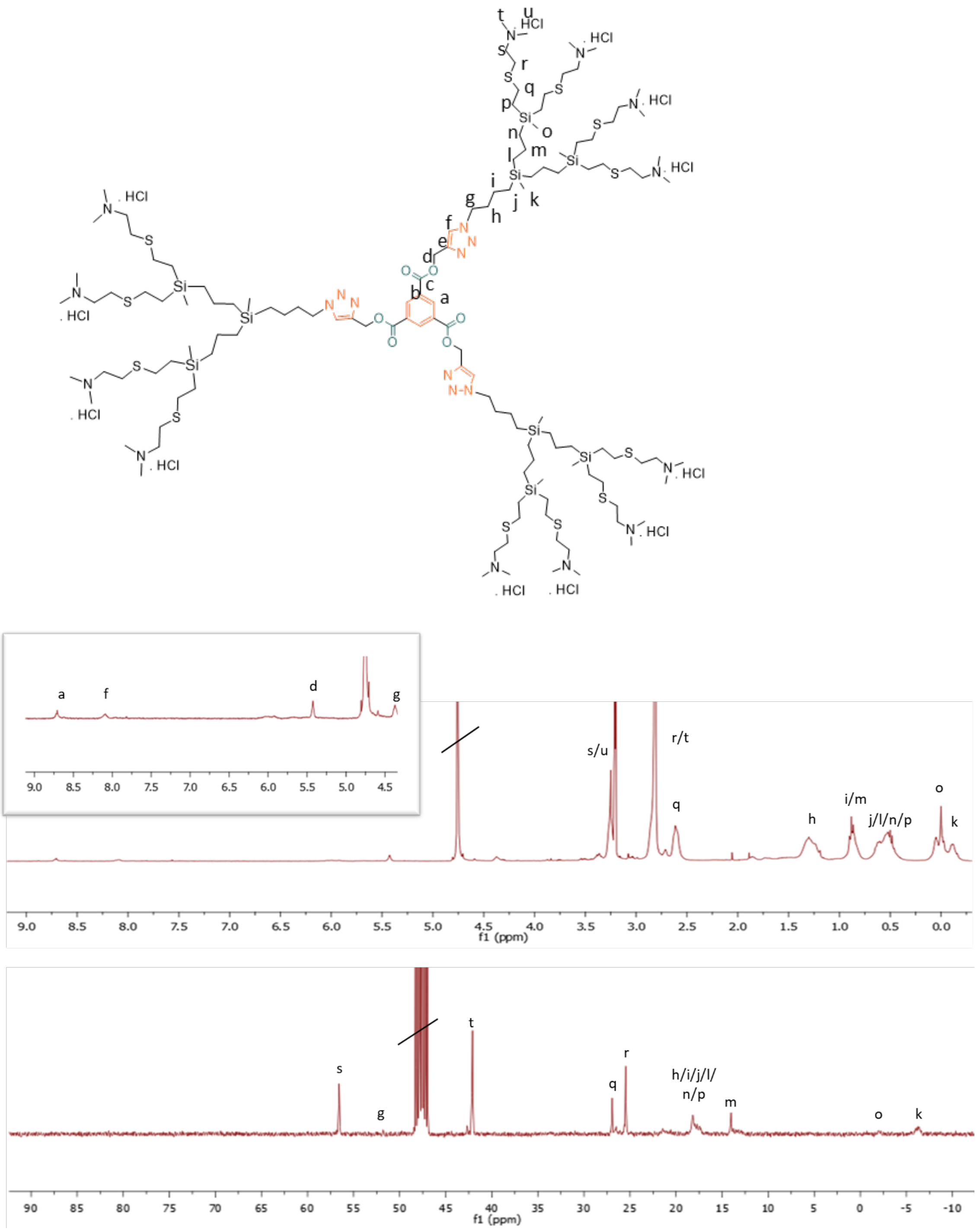

Figure S3. ^{1}H and ^{13}C NMR spectra of dendrimer **(2)** in MeOD.

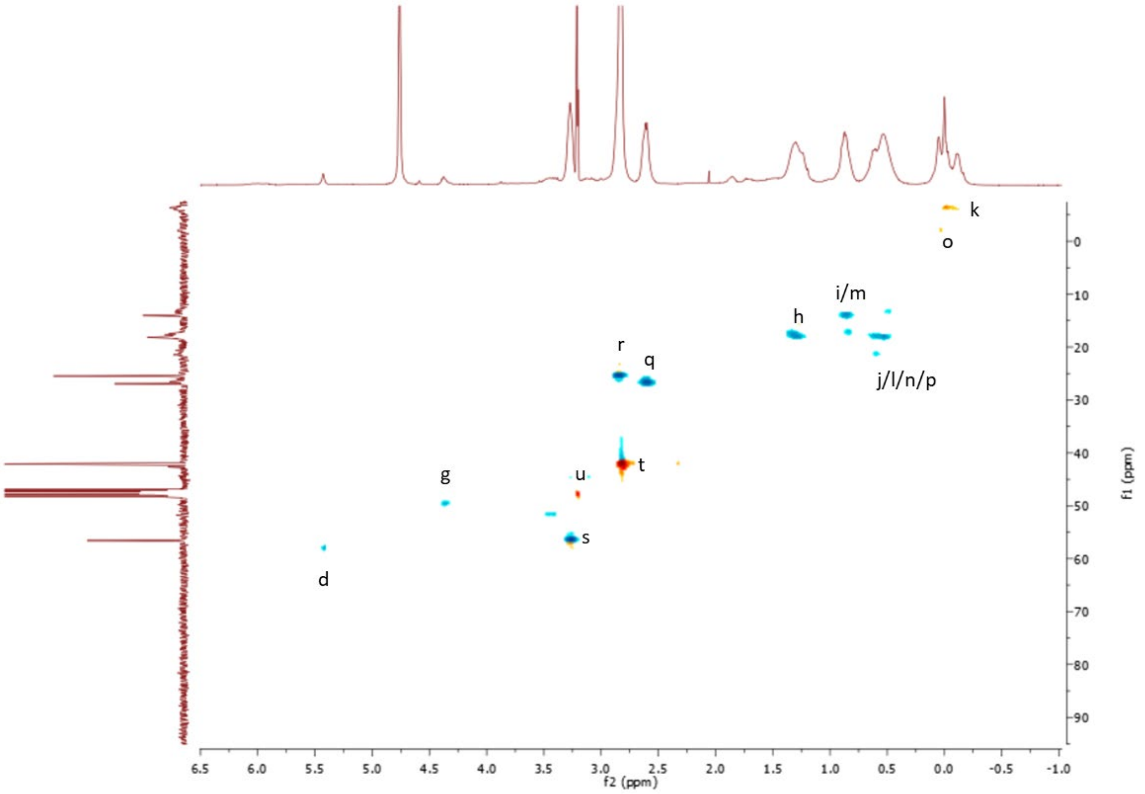

Figure S4. ^{1}H-^{13}C HSQC spectrum of dendrimer **(2)** in MeOD.

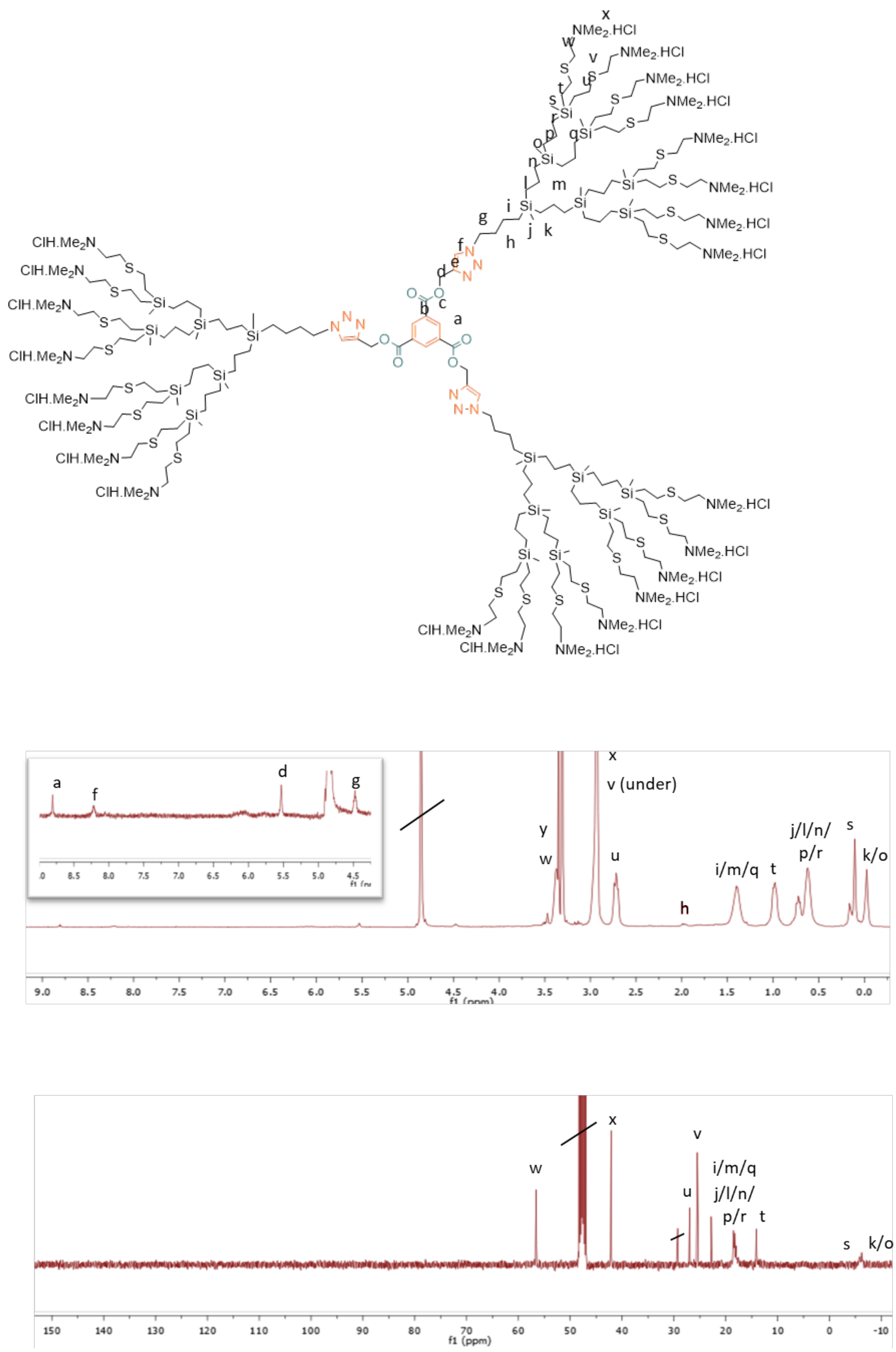

Figure S5. ^{1}H and ^{13}C NMR spectra of dendrimer **(3)** in MeOD.

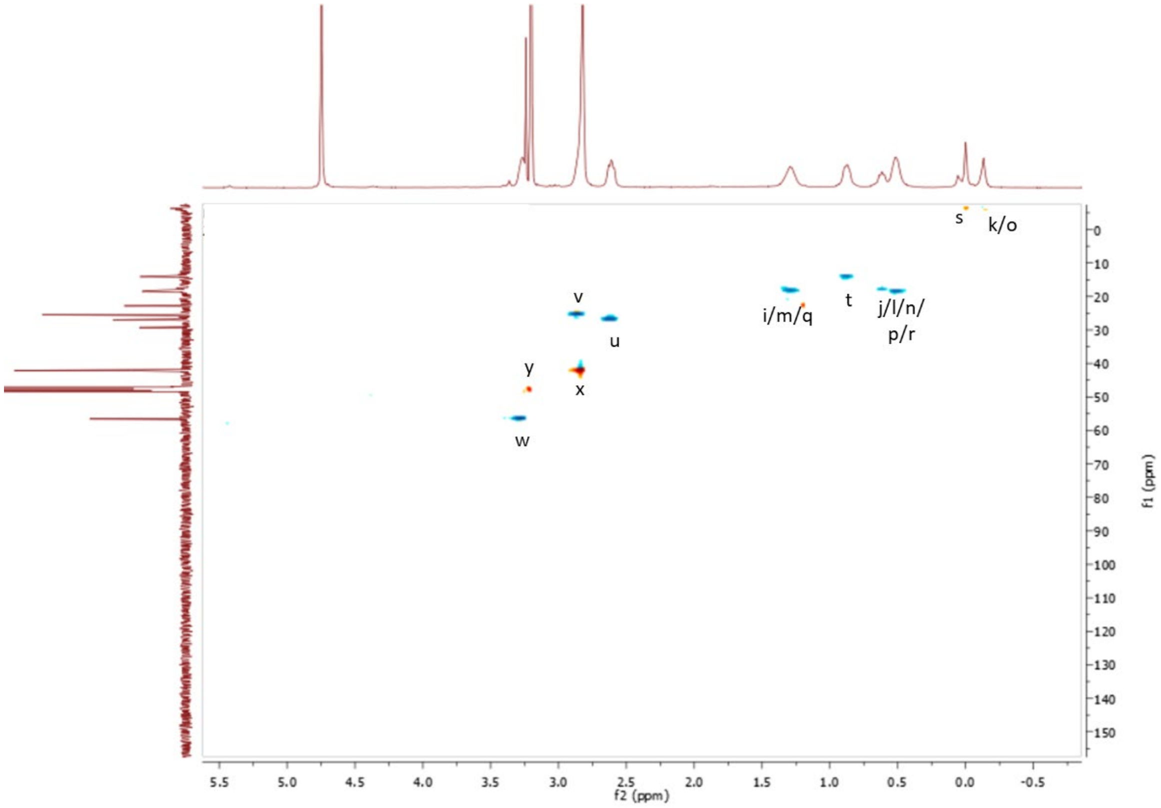

Figure S6. ^{1}H-^{13}C HSQC spectrum of dendrimer **(3)** in MeOD.

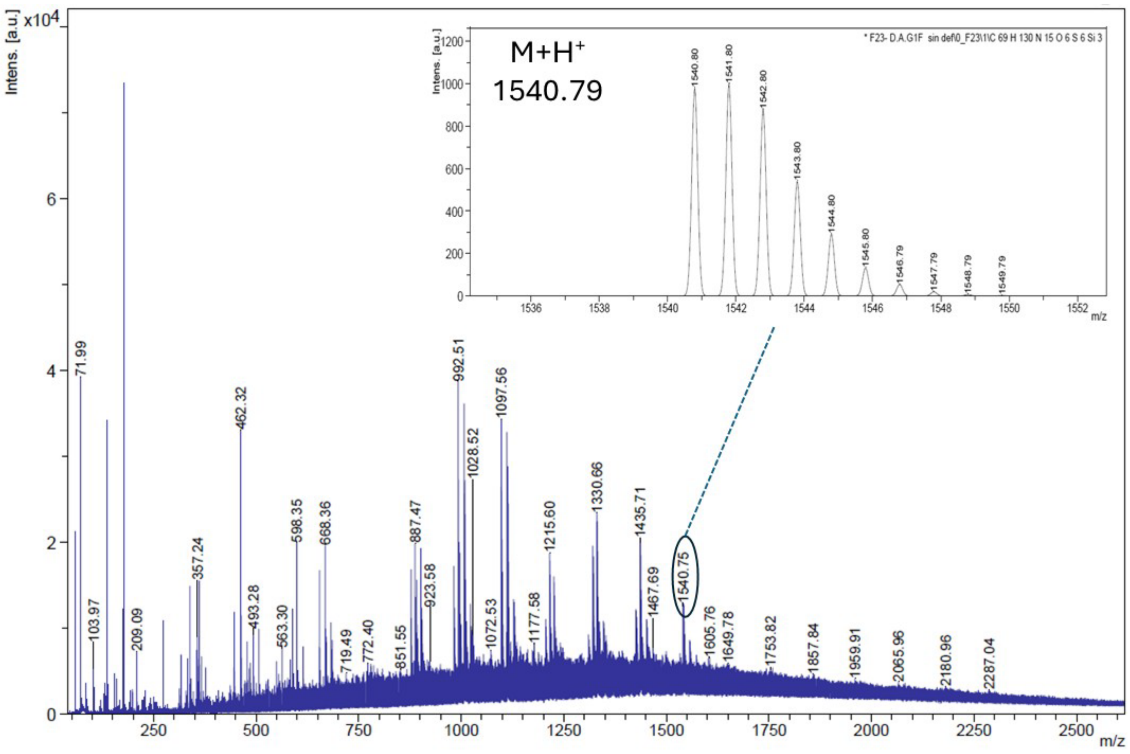

Figure S7. MALDI-TOF spectra of dendrimer **(1)** in DHB matrix.

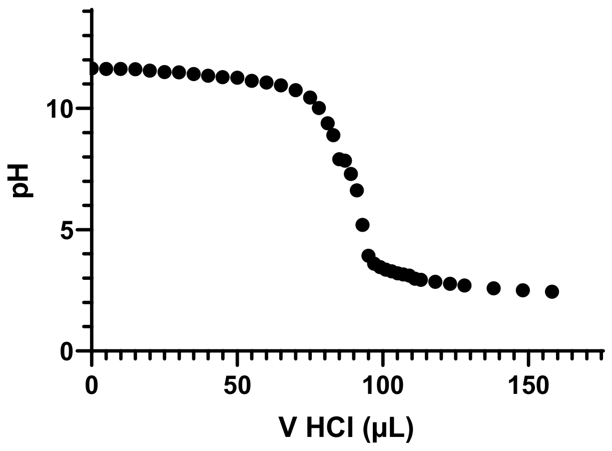

Figure S8. Potentiometric study of dendrimer **(1)** in water.

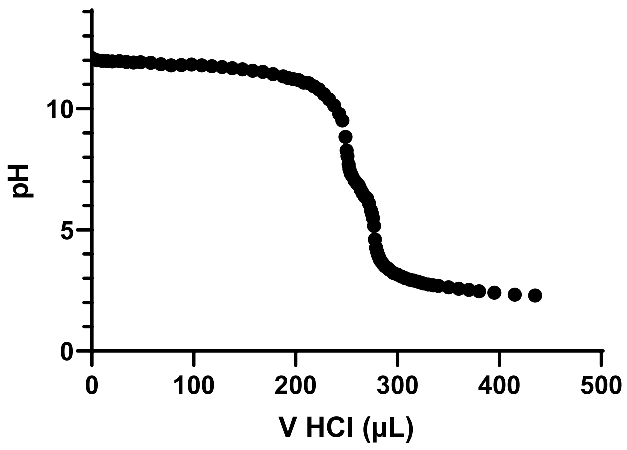

Figure S9. Potentiometric study of dendrimer **(2)** in water.

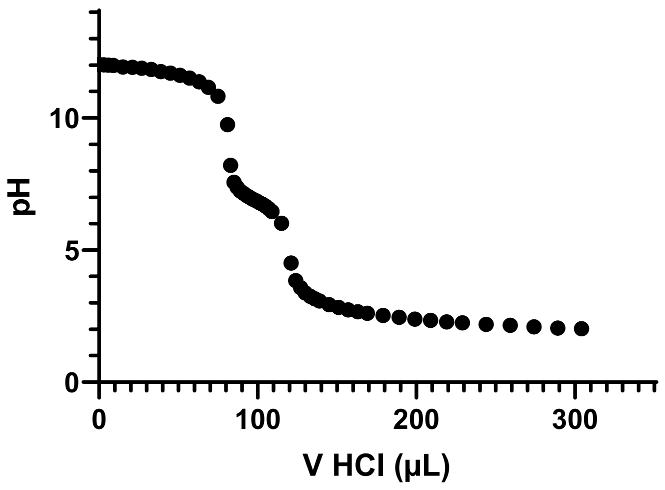

Figure S10. Potentiometric study of dendrimer **(3)** in water.

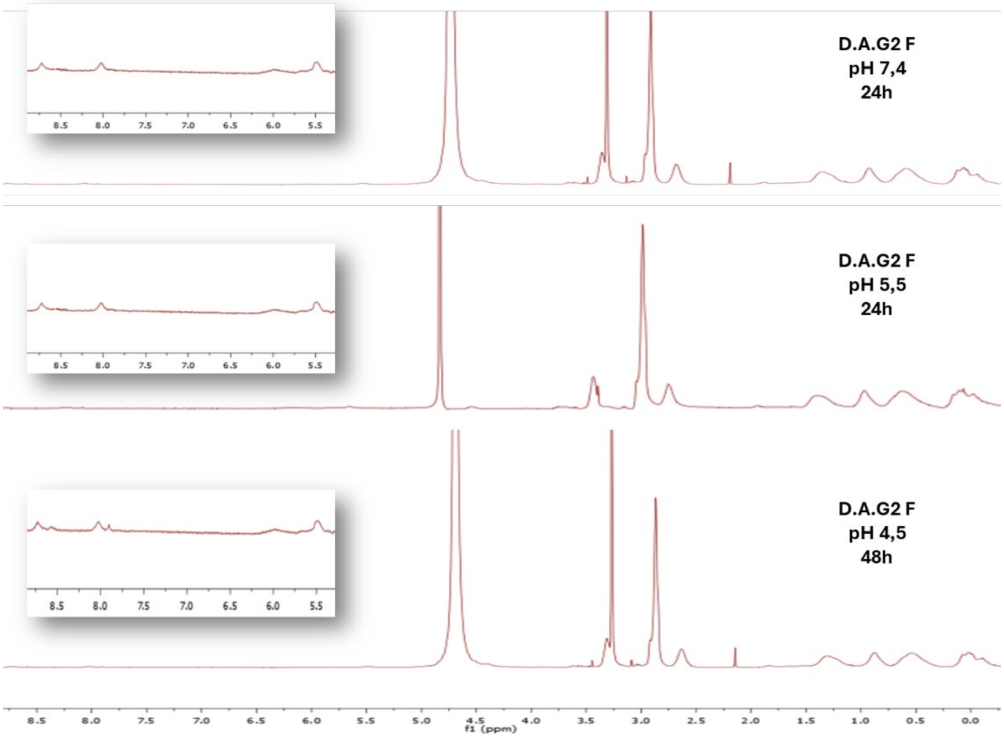

Figure S11. Degradation study of dendrimer **(2)** at different pH.

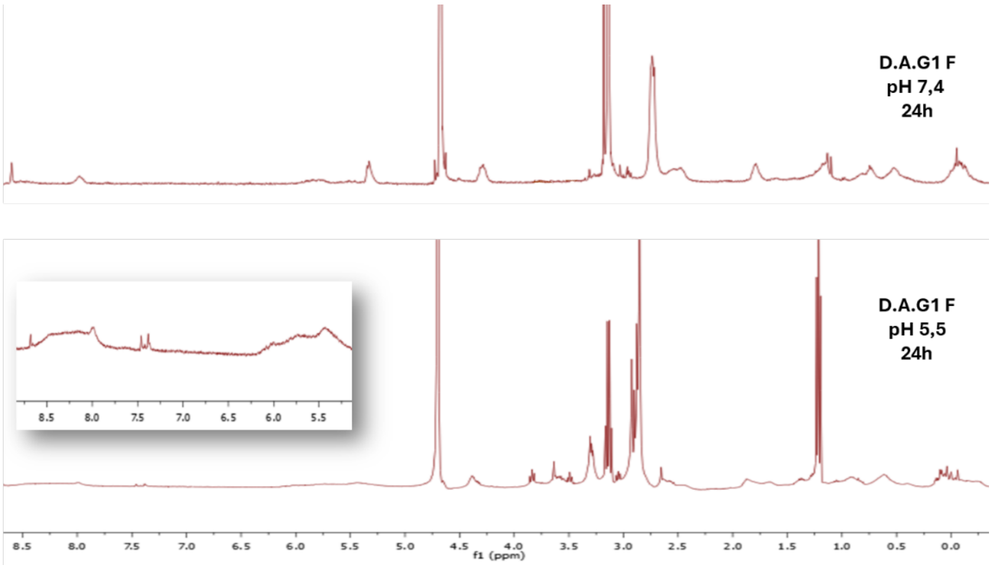

Figure S12. Degradation study of dendrimer **(1)** at different pH.

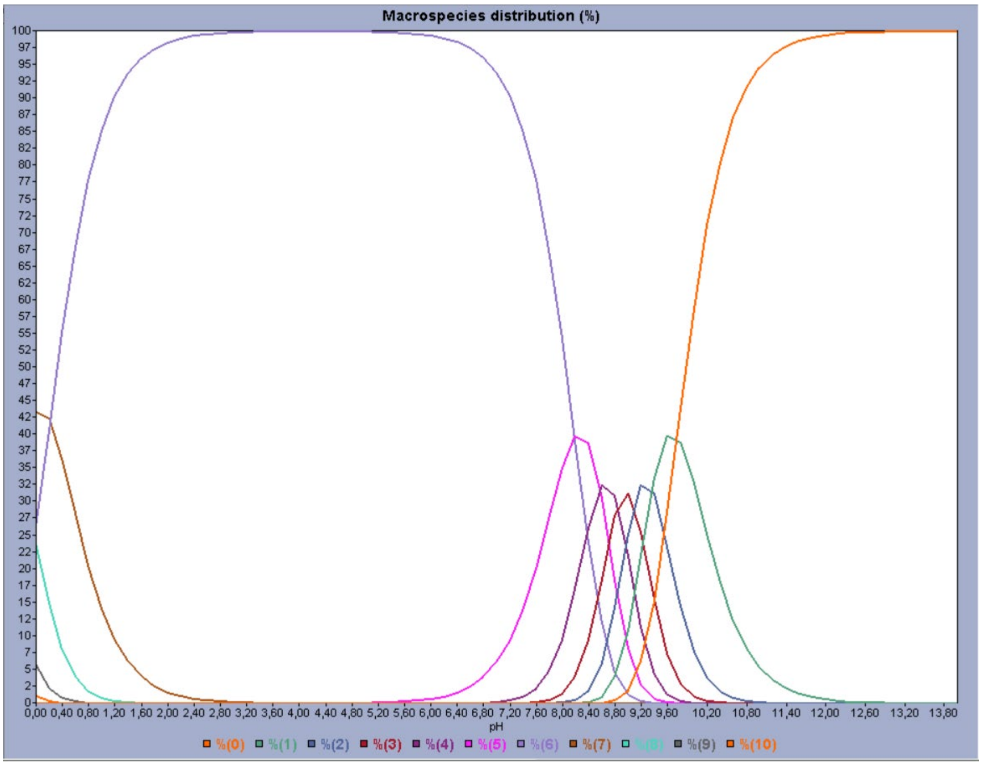

Figure S13. Macrospecies calculation of dendrimer **(1)** with MarvinSketch 22.7.

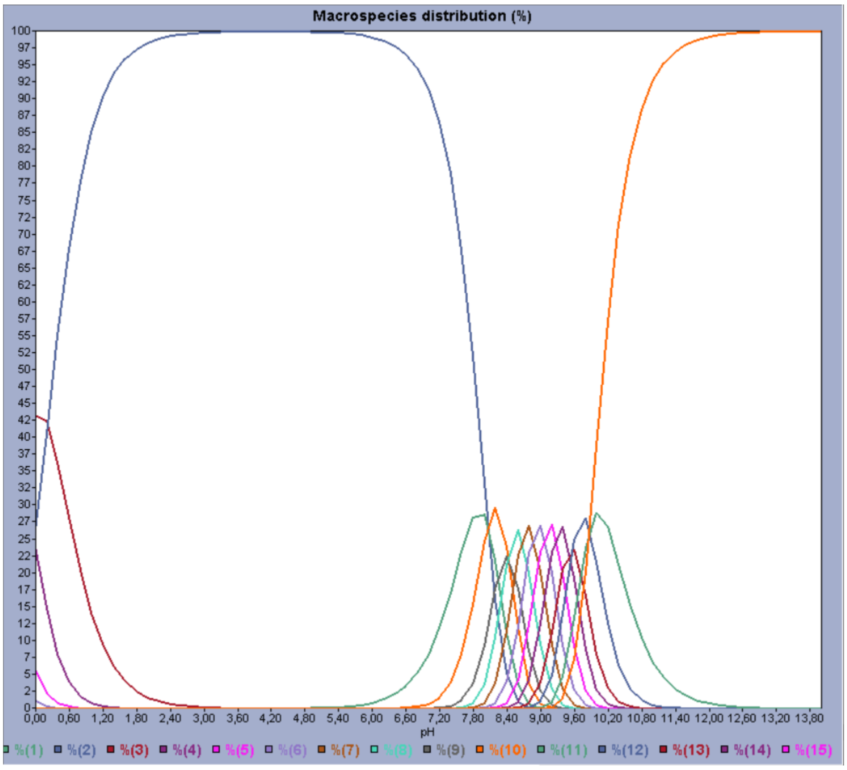

Figure S14. Macrospecies calculation of dendrimer **(2)** with MarvinSketch 22.7.

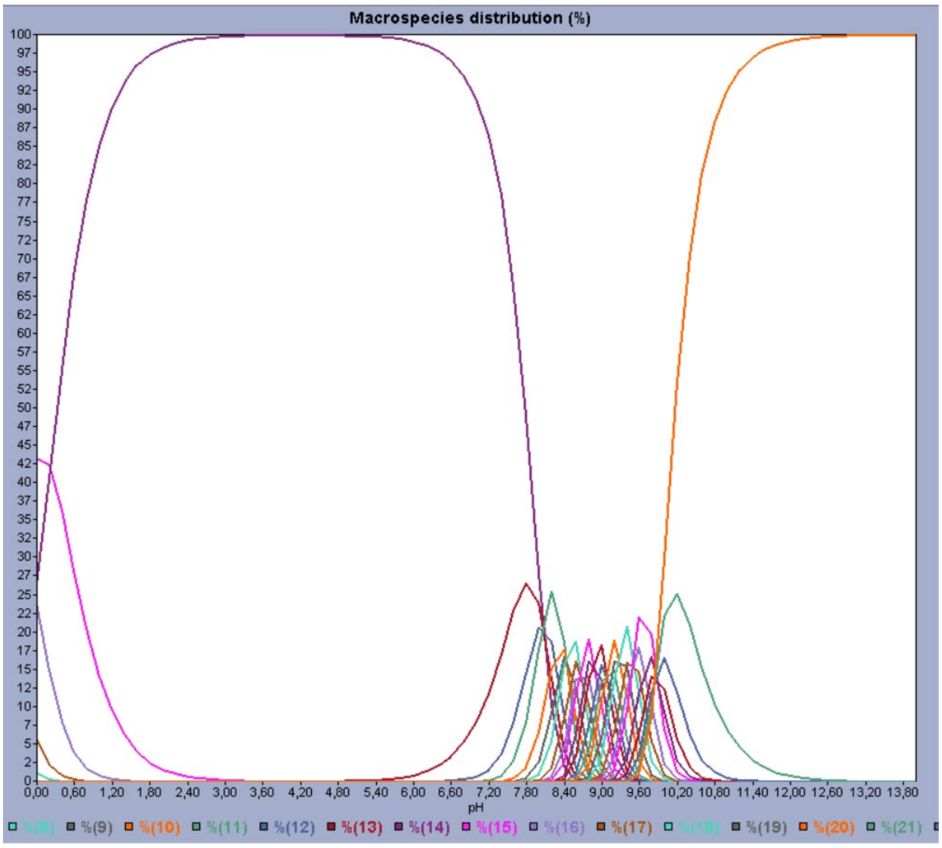

Figure S15. Macrospecies calculation of dendrimer **(3)** with MarvinSketch 22.7.

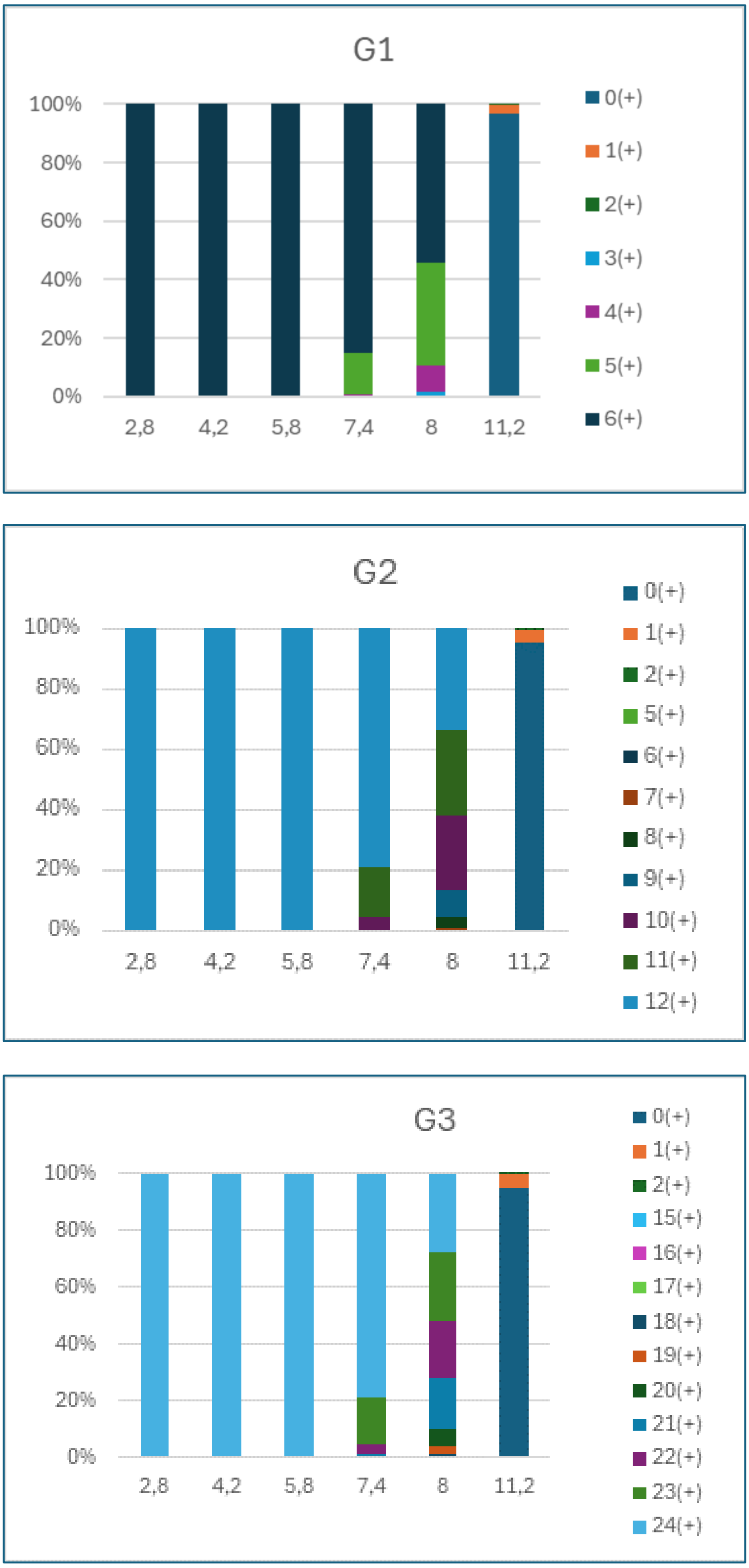

Figure S16. Comparative macrospecies distribution (%) at selected pH values (2.8, 4.2, 5.8, 7.4, 8.0 and 11.2) for dendrimers **1-3**.

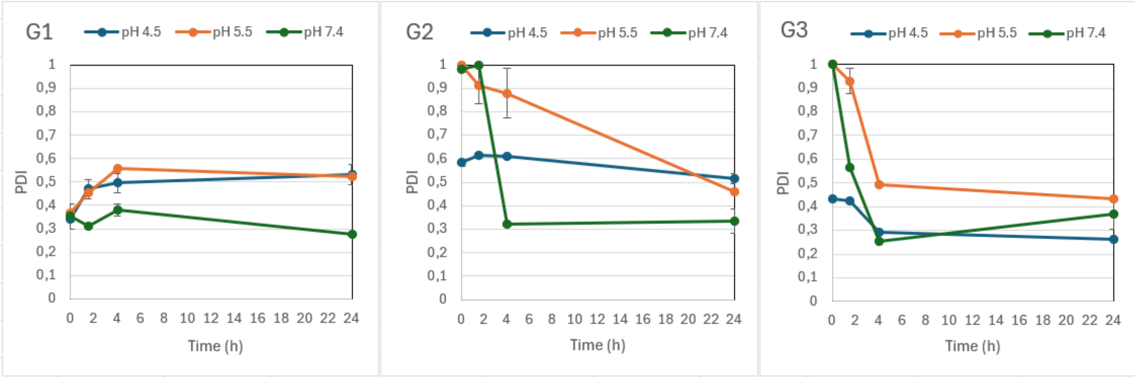

Figure S17. Evolution of PDI over time for dendrimers **1-3** at different pH.

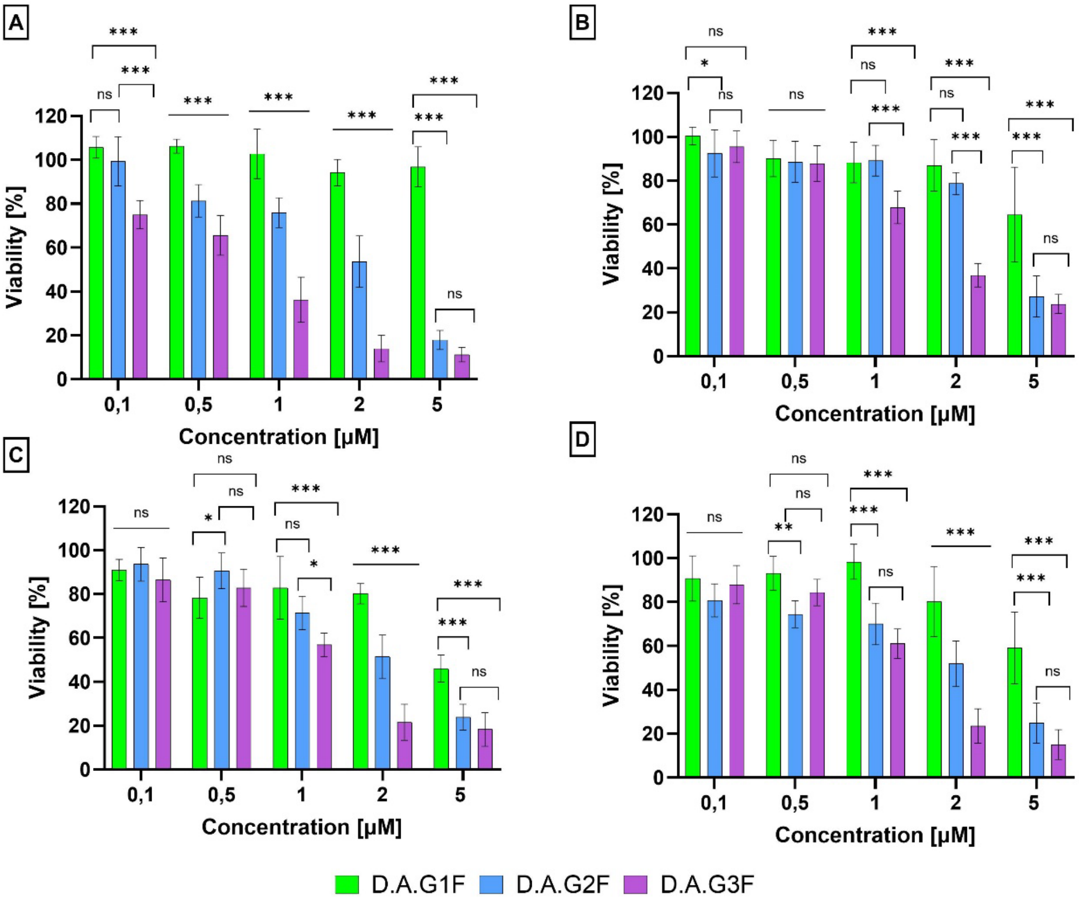

Figure S18. HEK-293 cell viability after 24h incubation with G1-G3 dendrimers at different pH.

[A]: pH=4.5; [B]: pH=5.5; [C]: pH=7.4; [D]: pH=10. Results presented as mean +/- SD, n = 3, *$p \leq 0.05$; **$p \leq 0.01$, ***$p \leq 0.001$, n=3.
